# DFT and TD-DFT Investigation of a Charge Transfer Surface Resonance Raman Model of N3 Dye Bound to a Small TiO_2_ Nanoparticle

**DOI:** 10.3390/nano11061491

**Published:** 2021-06-04

**Authors:** Ronald L. Birke, John R. Lombardi

**Affiliations:** Department of Chemistry and Biochemistry, The City College of the City University of New York, 160 Convent Avenue, New York, NY 10031, USA; jlombardi@ccny.cuny.edu

**Keywords:** Raman, surface enhance Raman scattering, charge transfer, surface geometry, UV-VIS, DSSC, N3 and related Ru bipyridyl dyes

## Abstract

Raman spectroscopy is an important method for studying the configuration of Ru bipyridyl dyes on TiO_2_. We studied the [Ru(II)(4,4′-COOH-2,2′-bpy)2(NCS)2)] dye (N3) adsorbed on a (TiO_2_)5 nanoparticle using Density Functional Theory, DFT, to optimize the geometry of the complex and to simulate normal Raman scattering, NRS, for the isolated N3 and the N3–(TiO_2_)5 complex. Two configurations of N3 are found on the surface both anchored with a carboxylate bridging bidentate linkage but one with the two NCS ligands directed away from the surface and one with one NSC tilted away and the other NCS interacting with the surface. Both configurations also had another –COOH group hydrogen bonded to a Ti-O dangling bond. These configurations can be distinguished from each other by Raman bands at 2104 and 2165 cm^−1^. The former configuration has more intense Normal Raman Scattering, NRS, on TiO_2_ surfaces and was studied with Time-Dependent Density Functional Theory, TD-DFT, frequency-dependent Raman simulations. Pre-resonance Raman spectra were simulated for a Metal to Ligand Charge Transfer, MLCT, excited state and for a long-distance CT transition from N3 directly to (TiO_2_)5. Enhancement factors for the MLCT and long-distance CT processes are around 1 × 103 and 2 × 102, respectively. A Herzberg–Teller intensity borrowing mechanism is implicated in the latter and provides a possible mechanism for the photo-injection of electrons to titania surfaces.

## 1. Introduction

**Figure 1:**
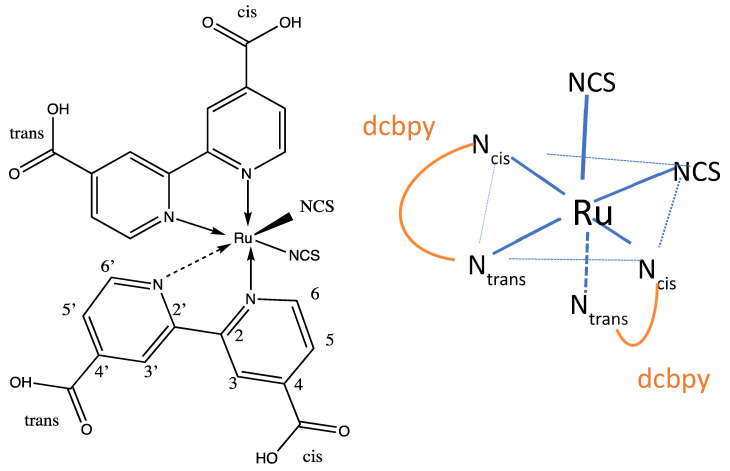
The chemical structure Ru(NCS)_2_(dcpby)_2_ called N3 and sketch of its structure.

The study of photoinduced processes at semiconductor-molecule interfaces has received much attention in the scientific literature because of its importance in both photo-catalytic and solar energy conversion processes. Chief among these processes is photoinduced electron transfer at nanostructured titanium dioxide nanoparticle (NP) surfaces sensitized by adsorbed dye-molecules. This process is the basis of the dye-sensitized solar cell (DSSC), first characterized by Grätzel and O’Regan [1]. An important aspect of the properties of these solar cells is the nature of the adsorption geometry of the dye molecule on the surface of the NP TiO_2._ The bonding of the adsorbed dye to Ti atoms at the titania surface affects the efficiency of the solar energy conversion process through both the injection time of electron transfer and changes in the conduction band structure at the surface of the TiO_2_ [2,3]. Although many new sensitizer dyes have been studied to improve solar conversion efficiency [4], the prototype dye [2] for studying DSSCs has been cis-di(isothiocyanate)di(2,2′-bipyridine-4,4′-dicarboxylic acid)Ru(II) or [Ru(NCS)_2_(dcbpy)_2_], known as N3 whose structure is shown in Figure 1. Neutral N3 in the solid form has all four carboxylates protonated but dissociates in water. A related dye is the N719 salt where two carboxylates in N3 have been deprotonated and tetrabutylammonium groups (TBA^+^) give charge neutrality for the solid. In aqueous solution at pH ≥ 1.5 both compounds form the N3 dianion [5]. The Raman spectroscopy of N3 absorbed on a small TiO_2_ nanoparticle by quantum mechanical electron structure methods is the subject of investigation in this paper. We will show that this model is able to give the most important binding geometry of N3 on TiO_2_

Figure 1 displays a sketch of the pseudo-octahedral complex on the right showing the nitrogen atoms of the pyridyl rings and carboxyl groups of dicarboxylbipiridyl [dcbpy] which are cis and trans to the isothiocyanate ligands. There is a large number of studies of N3 and N719 dyes adsorbed on titania surfaces, and we review in some depth the pertinent literature related to Raman and Optical spectroscopy, and the dye-nanoparticle structure.

### 1.1. Raman and Infrared Studies

Both infrared and Raman vibrational spectroscopy methods are important experimental techniques for examining the structure of adsorbed molecules like N3 on TiO_2_. Since normal Raman scattering is a relatively weak effect, some type of enhancement mechanism is necessary to obtain well-developed spectra for molecules on semiconductor surfaces. Indeed, adsorbed molecules on TiO_2_ nanoparticles have been shown experimentally to support Surface Enhance Raman Scattering (SERS) via a charge transfer (CT) mechanism [6,7], i.e., CT-SERS. For the TiO_2_-molecule system, it should be noted that a significant electromagnetic plasmonic field enhancement is unlikely as would be the case on metal substrates. For molecules on semiconductor (SC) surfaces, Raman enhancement mechanisms could involve either a solely molecular resonance Raman process for molecules which absorb light in the visible or a long-distance CT resonance mechanism for which the excitation occurs between the molecule and SC [8,9]. We will illustrate that these two photoinduced CT mechanisms are possible for N3 adsorbed on TiO_2_ with molecular resonance from Franck-Condon scattering and long-distance CT between N3 and the TiO_2_ nanoparticle from Herzberg-Teller scattering [8,9]. 

CT-SERS can be established as the enhancement mechanism [6] for adsorbed 4-mercaptobenzoic acid(4-MBA) on nanoparticle TiO_2_ where a molecular resonance is not possible. Another possible photoinduced CT resonance mechanism for the TiO_2_-molecule interface has been proposed [7] involving direct photoinduced charge transfer from the molecule ground state directly to the solid support without involving a Lowest Unoccupied Molecular Orbital, LUMO, on the molecule. This was called a Surface Enhanced Resonance Raman Scattering, SERRS, mechanism which should be distinguished from that of SERRS on metal substrates which involves molecular resonance in the molecule and the local surface plasmon resonance (LSPR) electromagnetic field enhancement from the metal substrate. Recently enhancement factors of 1.86 × 10^6^ and 1.3 × 10^6^ have been observed for 4-mercaptobenzoic acid on two-dimensional amorphous titania nanosheets [10] and for crystal violet on a nanofibrous three-dimensional TiO_2_ network [11], respectively. 

Of course, even higher enhancements can be obtained on SERS metal substrates like Ag and Au. A SERRS study of Ru dyes N719, N749, and Z907 was made on Au nanopeanuts and Au/Pt/Au nanorasberries [12]. This molecular resonance is due to Ru metal to ligand charge transfer, MLCT, which dominate the visible absorption spectra of the Ru bipyridyl dyes. An interesting feature of this study was the enhancement of the band at 2149 cm^−1^ from isothiocyanate indicating that N719 absorbs on Au via the sulfur end of the two NCS groups which are cis to each other. In an earlier study, Perez León obtained the spectra of N719 on Ag and Au colloidal nanoparticles in water, ethanol, and acetonitrile and assigned the bands in the different solvents [13]. N719 has also been studied on nanoporous Au surfaces [14]. The above studies show the difference between normal Raman scattering (NRS) spectra compared with resonance Raman scattering (RRS), SERS, and SERRS spectra on Ag and Au NPs.

Various types of surface Raman studies of N3 or N719 dyes have also been made on Ag film substrates which contain TiO_2_ layers, such as Ag@TiO_2_ core shell NP structures where the dye is on the titania surface [15,16]. Excitation at 532 nm includes both an intramolecular resonance and the LSPR enhancement [15]. Potential-dependent studies indicate that NCS is interacting with the TiO_2_ surface through the S atom. Also, the direct photoinduced CT from the Highest Occupied Molecular Orbital, HOMO, of N719 to the TiO_2_ conduction band is indicated [16]. Bing Zhao and coworkers [17] investigated Ag/N719/ TiO_2_ sandwich systems excited at 532, 633, and 785 nm with N719 adsorbed to Ag NPs via the isothiocyanate groups. A similar study was made for Ag/N3/TiO_2_ and TiO_2_/N3 systems [18]. Here the 1366 cm^−1^ band indicates that N3 is connected to TiO_2_ via the carboxylate group of the bipyridine groups. Two configurations of adsorbed N3 on TiO_2_ particles are proposed. One solely bound by two trans carboxylates groups and another bound with one cis carboxylate group and one cis isothiocyanate group. 

**Figure 2:**
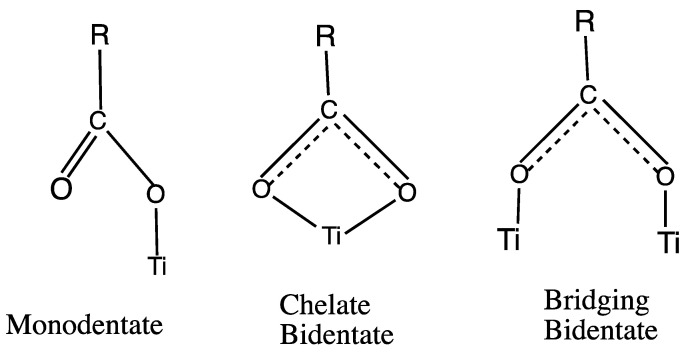
Three types of surface bonding to Ti atoms on titania surfaces.

Vibrational spectroscopy without SERS metal enhancements on clean TiO_2_ surfaces has also been used extensively to examine the N3 class of Ru sensitizer dyes with the goal of understanding how these dyes are coordinated to the surface of TiO_2_ through the carboxylate group. The three types of possible surface coordination which have been discussed in the literature are shown in Figure 2. One of the first studies with Raman and Infrared, IR, spectroscopy of N3 and its deprotonated forms on nanocrystalline titania was made by Finnie et al. [19]. They ruled out the monodentate configuration and concluded that N3 attaches to the TiO_2_ surface via either a bidentate chelate or bridging bidentate coordination (Figure 2) based on an empirical correlation in the literature [20] between the splitting of symmetric and asymmetric stretching vibrations for ionic carboxylate and N3 adsorbed on TiO_2_. Also, each N3 molecule was considered to bind to the surface with two coordinating carboxyl groups [19]. Bands in solid N3, N719, and N712 have also been assigned with Fourier Transform Infrared, FTIR in the photoacoustic mode [21]. For these dyes on TiO_2_ films, Attenuated Total Reflectance, ATR-FTIR, spectra were also obtained.

In a later Raman study, well-defined spectra were obtained by Greijer et al. [22] from the anode of solar cells made by soaking nanostructured TiO_2_ with N719 dye. In their work the emphasis was on the reaction of iodine with the isothiocyanate groups of the dye. The study of Shoute and Loppnow [23] obtained resonance Raman spectra at 514.5 nm excitation for free N3 in dimethyl sulfoxide, DMSO, solution and for N3 adsorbed on colloidal nanoparticles of TiO_2_ dissolved in DMSO. The dye was considered to be covalently bound to the Ti(iV) surface ions by the carboxylate groups on the bipyridine ligand. The resonance process of a metal-to-ligand charge transfer process, MLCT, was considered to occur by photoexcitation of N3 with oxidation of Ru(II) to Ru(III) and the formation of a bipyridine radical anion with subsequent fast electron transfer via carboxylate π* orbitals to the d orbitals of Ti of the conduction band. Shoute and Loppnow attribute the 1388 cm^−1^ band to a bridging or bidentate chelate linkage. The binding of Ru-bpy dye sensitizers on mesoporous TiO_2_ was again investigated with Raman and FTIR spectroscopies by Perez Leòn et al. [24]. For the N719, they concluded again that the anchoring occurs by either bidentate chelate or bidentate bridging coordination. This adsorption geometry for N719 is in agreement with Finnie et al. [19] and Nazeeruddin et al [21]. A later study by Lee et al. [25] concluded that additional consideration of surface groups of titania such as Ti-O, Ti-OH, and Ti-OH_2_ were necessary in considering the surface geometry of –COO^−^ and –COOH anchoring in dyes such as N719. 

In the above studies, the use of Raman and IR results could not distinguish between the bidentate chelate and the bridging bidentate modes of surface anchoring which stimulated further vibrational spectroscopy studies of N719 on TiO_2_ films. Schiffmann et al. [26] focused on the nature of the carboxylate binding geometry on TiO_2_ using ATR-FTIR experiments and DFT theoretical simulations. The IR results were compared with electronic structure calculations made with a slab model and a periodic boundary hybrid Gaussian and plane-wave (GPW) DFT methodology using CP2K software. It was found that none of the eleven vacuum optimized dye-TiO_2_ surface structures had the bidentate chelate mode. Our results, to be presented herein, also did not show this bidentate chelate geometry. 

The consideration from the above vibrational and DFT calculational studies of N3 like Ru dyes indicate that several adsorption configurations are possible and that they may coexist or can be interconverted. The Raman and IR spectra seem to rule out the monodentate ester configuration, but could not differentiate between the bridging bidentate or bidentate chelate forms [21,24]. The most definitive vibrational marker of chemisorption via carboxylate bonds for N3-like sensitizer dye is the band in 1370–1388 cm^−1^ region assigned to the symmetric COO^−^ stretch since it is found for N3 on the TiO_2_ film but not in solution. Raman simulations with our N3-(TiO_2_)_5_ model agree with this conclusion.

One of the first approaches to modelling the adsorption configuration of the N3 dye on TiO_2_ was to consider the known crystal structure of nanocrystalline anatase (101) in relationship to the determined X-ray crystal structure of N3 [27]. Several adsorption models were proposed based on carboxylate interaction with the anatase (101) cut surface. This paper is an important source of data for comparing bond distance and angles from DFT calculations with X-ray data for N3. 

### 1.2. Optical Studies, Theoretical Electronic Structure, and Charge Transfer Mechanisms

In order to elucidate Raman behavior of N3 and N719 dyes and indeed, the fundamental processes in dye-sensitized solar cells, it is important to model the UV-VIS absorption curves for the isolated dyes and the dyes adsorbed on model TiO_2_ NP surfaces. Fantacci and co-workers [28,29] carried out DFT geometry optimization and TD-DFT absorption spectra simulations for isolated N3 in vacuum and in ethanol. Persson and Lundqvist [30] introduced a model for N3 adsorbed on a (TiO_2_)_38_ 1.5 nm nanoparticle cut from (101) anatase and carried out optical absorption simulations. Full geometric optimization at the B3LYP level in vacuum were calculated with Ru, N, and S atoms using the LANL2DZ ECP basis sets while 6–31G* was used for H, C, O, and Ti atoms. The structure of this complex had two binding sites to the nanocrystal from carboxylates on the same bipyridine and was titled so that S atom end of the isothiocyanate was 3.5 Å from the surface. The optimized structure had two bridge binding anchoring groups. These calculations show that the first few excitations involve MLCT transitions to orbitals within a few tenths of an eV of the conduction band orbitals. 

This work was followed by several papers on electronic structure calculations from Grätzel and coworkers [3,31,32,33] with N719 dye also on (TiO_2_)_38_ nanocrystal model surfaces. In these papers, the structures of N719 and other related dyes adsorbed on TiO_2_ slab models were optimized by the Car-Parrinello (CP) method. With two anchoring carboxylate groups, a structure called B with two protons transferred to the surface was energetically favored [3,31] over the structure A where the protons were retained on the dye. A most interesting results is that on the low energy structure B [31], there are mixed-dye TiO_2_ states. The authors conclude this strong coupling suggests ultrafast electron injection rates. These studies illustrate that mixed states could be considered as indicating a direct electron transfer from the Ru-NCS HOMO in the TiO_2_ band gap to empty conduction band orbitals of the TiO_2_ cluster [31,33]. In fact, a charge density difference plot shows this direct electron transfer [33]. Such a direct excitation mechanism is also indicated from the natural transition orbital (NTO) hole-electron plot we will present herein.

For DSSCs there are a number of kinetic pathways which are important [34]. However, for the surface Raman enhancement process for Ru sensitizer dyes on the TiO_2_ surfaces, it is the mechanism of the photo-induced electron transfer, ET, which is of primary interest. Such a mechanism could change with a change in the excitation energy of the exciting light. Two possible types of ET mechanisms for the dye-TiO_2_ interface have been delineated in the literature [35,36,37]. Person et al. [35] described the ET mechanism on the basis of INDO semiempirical calculations for a dye-TiO_2_ model. In both types of ET, the ground state of the dye lies in the gap between the valence band (VB) edge and the conduction band (CB) edge. In scheme 1, the photoexcitation transfers the electron from the dye HOMO to an antibonding unoccupied intermediate state above the conduction band. It is from this level that the ET injection occurs to the Ti(3d) levels of the conduction band, presumably via electron tunneling. In scheme 2, the photoexcitation transfers the electron from the dye HOMO directly to the conduction band edge, without going through an intermediate excited state of the dye. This latter scheme is facilitated by a strong substrate-adsorbate interaction. For surface Raman scattering at TiO_2_, both schemes 1 and 2 are possible and would engender a different enhancement process: a molecular resonance Raman process (Franck-Condon scattering) in scheme 1 and a long distance CT from dye-to-TiO_2_ (Herzberg-Teller scattering) in scheme 2, where intensity borrowing is possible [8,9]. For DSSCs these electron injection schemes were called Type-I (two-step) and Type-II (one-step) pathways, and the Ru(II) dyes bound to TiO_2_ through carboxylates were classified as Type-1 [36]. This classification is certainly dependent on whether the energy of the exciting light promotes the electron to a state on the dye or to the Ti(3d) unoccupied state of titania. Thus, for excited state S18 in the model of De Angelis et al. [33], N719-(TiO_2_)_38_ follows a scheme 2 (Type-II) one-step electron injection. However, early experimental results were considered to show the two-step mechanism (Type -I) [38]. The injected electron rate is considered to be controlled by the Franck-Condon overlap of vibrational states of reactant and product. Another study of dyes on colloidal TiO_2_ [39] using Stark emission spectroscopy found evidence for both types of mechanisms indicating the possibility of injection from a state with significant interfacial CT character. Our excited state hole-electron iso-surface plot also suggests such a one-step direct CT process is possible. 

The two-step mechanism has been investigated in a theoretical study of resonance Raman scattering, RRS, at a dye-semiconductor surface [40]. This treatment is based on an Anderson-Newns type Hamiltonian with a continuum of semiconductor states approximated by a set of discrete states with uniform energy in terms of Legendre polynomials. It was found that for this model intensities of Raman active modes in the isolated dye are de-enhanced on the surface because of the population decay of the dye excited state by the electron injection process. A one-step mechanism would not be subjected to this decay, but here one would expect to observe a red shifted optical spectrum from a CT state with a dye cation D^+^ and the electron e^−^ in the conduction band. However, if the oscillator strengths for excitation to such CT states are very weak, they would not be observable in the absorption spectrum. In fact, both the direct one-step and intermediate two-step electron transfer mechanisms for different configuration of xanthene molecule complexes on the same TiO_2_ surface has been proposed [41].

In several ultrafast experiments, typically at 400 nm excitation [42], the two-step process has been assumed with excitation to the MLCT state of N3 with the electron in the π* orbital of dcbpy followed by injection of the electron to the semiconductor conduction band. These authors conclude that a direct CT transition is unlikely from Ru dye to TiO_2_ because of the lack of overlap between Ru dye and TiO_2_ orbitals. However, our calculations show some oscillator strength for such a transition.

Other evidence for a direct charge-transfer mechanism from adsorbed Ru dyes comes from Raman spectra with enhanced phonon modes of TiO_2_ NPs [43,44,45]. With N719 on the TiO_2_, there is enhancement of the phonon modes with 514.5 nm excitation which is attributed to the charge transfer process [44]. Fourth order coherent Raman spectroscopy with 20 fs pulses was also used to examine N3 dye on TiO_2_ (110) surfaces [45]. This technique examines the interfacial region and thus surface modes of the TiO_2_ can be observed. The surface mode at 100 cm^−1^ and the E_g_(1) mode at 146 cm^−1^ where found to be strongly enhanced. Several enhancement mechanisms were proposed including the Herzberg-Teller CT-SERS mechanism of Lombardi and Birke [8,9] where the ground state of N3 is located in between the valance band and conduction band and there is direct CT from this state to the conduction band with intensity borrowing from a strongly allowed transition of the dye.

### 1.3. TiO_2_ Nanoparticle Models

Many studies in the literature have been made with models N3 or N719 molecule adsorbed on a TiO_2_ NP complex using a (TiO_2_)_38_ model for the titania nanoparticles [3,31,32,33,35]. Even larger TiO_2_ model NPs have also been explored [46,47]. An important parameter for assessing the adequacy of the (TiO_2_)_n_ clusters is the HOMO-LUMO (HL) gap, which is an estimate of the band gap between the valence and conductance band edges. With B3LYP/LANL2DZ, for n between 16 and 60, band gaps between 4.55 and 4.94 eV were determined [48]. In another study [47], B3LYP and a variety of basis sets were used to calculate the HL gap for n = 1 to 68. Because we are calculating static and time-dependent Raman with TD-DFT, a large nanocluster model was found impractical for our calculations. We have thus used a much smaller (TiO_2_)_n_ cluster with n = 5. For this nanocluster, our B3LYP/6-31+G(d)/LANL2DZ result for the HOMO-LUMO (HL) gap is 4.45 eV which is within 0.1 eV of calculated values with a B3LYP/6-311 + G(2df,p) calculation for the (TiO_2_)_5_ cluster C in the paper of Lundqvist et al. [47]. Furthermore, HL band gap for n = 5 found herein is consistent with the HL band gap for much larger nanoclusters up to n = 68 [47]. This HL band gap is of the order of 1.5 eV larger than the experimental band gap or a bulk band gap calculated using a B3LYP and a periodic calculation. This increased band gap found for nanoclusters up to about 1nm in size has been attributed to a quantum size effect (confinement). Furthermore, there was a good quantitative fit of excitations energies for TD-B3LYP compared with the Equation of Motion Coupled-Cluster, EOM-CC, method [48]. Thus, based on the reasonable results for B3LYP properties of the (TiO_2_)_5_ NP for ground state and excited state calculations, we have used this size, n = 5, cluster with N3 adsorbed on the surface to simulate our Raman calculations.

## 2. Computational Methods and Models

Various forms of N3 dye, cis-Ru(II)-(dcbpy)_2_(NCS)_2_, either isolated or as a complex bound to a (TiO_2_)_5_ nanoparticle were used for spectral simulations. Calculations were made with DFT and TD-DFT using the Gaussian 16 software package [49] for geometric optimization, IR and Raman frequency, and time-dependent excited state optical and Raman calculations. All ground and excited state frequency calculations were made in the harmonic approximation. All calculations were made with the B3LYP hybrid GGA exchange-correlation density functional [50,51] with the 6-31+G(d) basis set for C, N, O, S, and H and with the LANL2DZ effective core potential (ECP) basis set for the Ru and Ti atoms. The B3LYP density functional has been used in most studies of dyes on TiO_2_ surfaces. UV-VIS absorption spectra were calculated for both gas phase (vacuum) and an ethanol solvent environment. For the ethanol calculations, the optimized structure and excitation spectrum were made with the SCRF default method of G16, which is the Polarizable Continuum Model (PCM) using the integral equation formalism variant (IEFPCM). The nature of the excited state transitions for Gaussian calculations was examined with Natural Transition Orbitals (NTO) [52] and with the charge-transfer distance index, D_CT_, and the amount of charge transferred index q_CT_ [53].

Normal Raman vibrational frequencies with G16 were computed at a stationary point by determining the second derivatives of the energy with respect to the Cartesian nuclear coordinates and then transforming to mass-weighted coordinates. In all cases, no imaginary frequencies were found. Pre-resonance frequency-dependent TD-DFT Raman vibrational calculations were made in G16 with the Coupled Perturbed Hartree Fock (CPHF) method. An empirical scaling factor of 0.970 was used to scale all normal mode wavenumbers.

To simulate Raman spectra with an N3–TiO_2_ nanoparticle complex, we have used N3 bound to a Ti_5_O_10_ nanoparticle_._ This model is based on the reaction of a single negatively charged N3, which is formed by the loss of a proton from one of the four COOH groups on neutral N3, forming a bridging bidentate bond to the neutral Ti_5_O_10_ nanoparticle. This single –COO carboxylate anchoring group of N3 forms a bridging bond to two Ti atoms of the Ti_5_O_10_ nanoparticle. The structure of Ti_5_O_10_ was cut from a much larger slab of an anatase polymorph. The initial structures of the N3–Ti_5_O_10_ complex with unidentate ester or bidentate chelate binding to a single Ti atom were found to always converge to the bridging bidentate structure on this Ti_5_O_10_ nanoparticle. Previous theoretical studies of dyes adsorbed on TiO_2_ polymorphs have used much larger nanoparticle models but typically with a much lower level basis set such as 3-21G(d) and did not include Raman simulations. These were mostly made for the study of excited states. However, for accurate vibrational Raman calculations, a higher order basis set is necessary. Geometric optimization followed by Raman vibrational simulation for N3 bound to larger TiO_2_ nanoparticle models with a B3LYP/6-31+G(d)/LANL2DZ calculation was found to be impractical. GaussView 6.0 [54], Chemcraft [55], and Multiwfn 3.8 [56] were used to view and analyze structures and spectra.

## 3. Results and Discussion

### 3.1. Optimized Geometry of Fully Protonated N3

**Figure 3:**
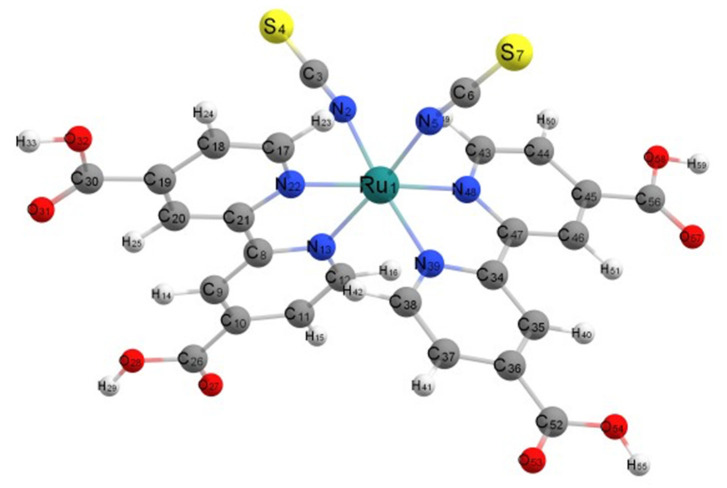
Optimized structure of neutral cis-N3 in vacuum.

The optimized geometry of all forms of N3 was found to be a pseudo-octahedral complex of Ru^2+^ as shown for the neutral species in vacuum in Figure 3. The Ru central atom is coordinated to six nitrogen atoms from two bipyridyl groups and two isothiocyanate ligands. Here, N3 optimizes to C_1_ symmetry; however, in ethanol, the structure optimized to C_2_ symmetry with B3LYP/6-31+G(d)/LANL2DZ. In these structures, as seen in Figure 1, the two thiocyanate anions, NCS^−^, are cis to each, and the remaining nitrogen atoms of the bipyridines binding to Ru(II) are either cis or trans to the thiocyanate ligands. One can also denote the carboxylate groups on the pyridine rings of bipyridine as either cis or trans to the isothiocyanates.

In order to validate the computational method, we compare our calculations for neutral N3 complexes to two other vacuum calculations in the literature. Since there can be differences in the bond distance between the same type of Ru–N bonds on the different bipyridyl rings, we have denoted them as rings A and B. All the computational calculations in Table 1 show hardly any difference between rings A and B for the same type of Ru–N bond. However, this is not true for the X-ray experimental values. Our calculations are similar to the recent calculation [57] that was made with B3LYP and a slightly larger basis set, 6-311G(d) with a small core relativistic ECP for Ru. The three optimized structures in vacuum for N3 in Table 1 show slightly larger bond distances than the X-ray data [27] for Ru-bpy bonds; however, solid-state packing forces could account for these differences. By and large, all of the calculations in the table can be considered a reasonable representation of the N3 structure. Interestingly, the calculation with the BPW91 density functional [28] was constrained to C_2_ symmetry, whereas our calculation in ethanol was optimized to C_2_ symmetry.

The data above is for the fully protonated N3. We have also preformed geometric optimization in vacuum for the N3 monoanion, which is deprotonated from one cis-carboxylate, N3^−^(cis), and for N3 monoanion, which is deprotonated from one trans-carboxylate, N3^−^(trans), and for the dianion N3^2−^ in which two cis-carboxylates are deprotonated, which are on different bipyridine rings. It should be pointed out that there is an approximate C_2_ axis that bifurcates the angle formed by the two isothiocyanate groups and that the molecule can be considered as having one long axis and two short axes. The long axis is from the end of one cis-carboxylate to the end of the other cis-carboxylate [27]. The short axes are from the end of one isothiocyanate groups to the end of the opposite trans-carboxylate, as shown in Figure 3. These calculations for the anion structures were used to examine the effect of molecular charge on the dipole moment. For N3(0), N3^−^(cis), N3^−^(trans), and N3^2−^(cis), the dipole moments are 12.62 D, 25.80 D, 15.56 D, and 59.68 D, respectively. For N3(0), the dipole moment is directed approximately along the C_2_ axis with the negative end between the NCS^−^ groups. For N3^−^(cis), the dipole moment is directed approximately along the long axis with the negative end on the deprotonated carboxylate. For N3^−^(trans), the much lower dipole moment makes an approximately 90^o^ angle with the C_2_ axis and is approximately in the plane formed by the two isothiocyanates with its negative end near the Ru and its positive end directed over the other trans-carboxylate, which is protonated. For N3^2−^, which is the same as the dianion of N719, the carboxylate groups are deprotonated on opposite sides of the long axis. Here, the dipole moment is also directed along the C_2_ axis. It is interesting to compare this dipole moment, 59.68 D, with that of the similarly deprotonated species N719 b [3], 28.0 D, where the dipole moment is also along the C_2_ axis. The difference is that in N719 b, the cis-carboxylate ions have Na^+^ counterions, which balance the charge and lower the magnitude of the dipole moment by more than 50%. With N3(0) and N3^−^(cis) in ethanol using the IEFPCM, self-consistent reaction field calculation, the dipole moments are 21.12 D and 38.50 D, showing the significant effect of solvation on increasing the electronic polarization. By and large, the direction and magnitude of the dipole moments follow chemical intuition.

### 3.2. Geometric Optimization of the N3-Ti_5_O_10_ Complexes

**Figure 4:**
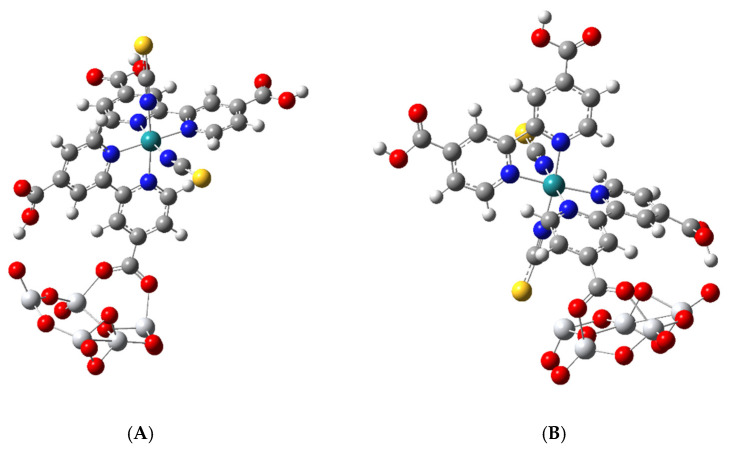
Optimized structures of two N3-Ti5O_10_^1−^ negatively charged singlet complexes. (**A**) Bridging bidentate structure with H-bond from a cis-carboxylate. (**B**) Bridging bidentate structure with a -NCS group bent toward the surface with H-bond from a cis-carboxylate.

**Figure 5:**
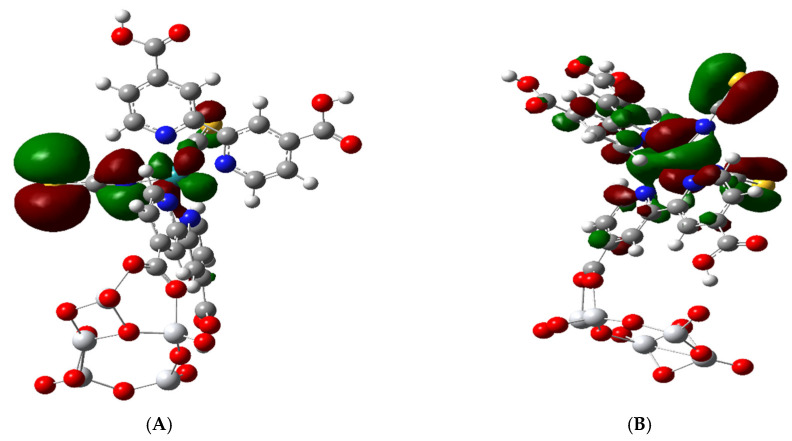
Filled molecular orbitals of the N3-(TiO_2_)_5_^−^. Right Side (**A**): HOMO-1 which is similar to the HOMO and to H-2 and H-3 MOs. Left Side (**B**): HOMO-7 which is similar to H-5 and H-6 MOs.

The optimized structure of two N3–Ti_5_O_10_ complexes is shown in Figure 4. Complex A was obtained by starting with the bridging bidentate structure, while complex B was obtained by starting with N3 bound by a monodentate ester linkage to one Ti atom. Both were optimized to the bridging bidentate structure through a trans-carboxylate group and both had a cis-carboxylate hydrogen-bond to a dangling O atom of the nanocluster; however, in complex B, one isothiocyanate is tilted toward the surface. This type of bridging anchoring was discussed as a possible arrangement for sensitizing dye on the (101) anatase surface where it was referred to as the B,C-type; see Figure 5 of reference [27]. The B-type is when a trans-carboxylate, which is on the so-called short molecular axis, binds to the surface, and the C-type is when cis-carboxylate, which is on the so-called long molecular axis, binds to the surface. Here, both complexes A and B in Figure 4 are anchored to the surface by the trans-carboxylate or B-type. For such a model anchoring geometry, 71 fragments in the Cambridge Structural Database gave average parameters of bridging Ti-O(dye), 2.03 Å; O–C, 1.26 Å; Ti–O–C, 135°; and O–C–O, 124°. The bond distances and bond angles for our two complexes have values remarkably close to these average values.

Thus, for complex A, we calculated values of bridging Ti-O(dye), 2.02 Å and 2.07 Å; O–C, 1.27 Å; Ti–O–C, 139°, O–C–O, 125°. For complex B, we calculated values of bridging Ti–O(dye), 2.04 Å and 2.09 Å; O–C, 1.27 Å; Ti–O–C, 129°, O–C–O, 126°. Our data indicate that one side of the bridging bidentate linkage is shorter than the other side. In the eleven structures of Schiffmann et al. [26], which are calculated for a slab model with 144 TiO_2_ units, the typical bond lengths are 2.08 Å for the bridging bidentate linkage and 2.02 Å for the monodentate bonds. It can be seen in Figure 4 that in both A and B complexes, the cis-carboxylate on the same bipyridine that binds to the surfaces through its trans-carboxylate forms a hydrogen bond to a terminal O atom of the nanocluster. The H-bond distance, C–O–H–O, is 1.76 Å for complex A and 1.47 Å for complex B. In B, the bond distance between the S atom of –NCS and Ti is 2.52 Å. The data for our optimized structure for complex A and B show good agreement between experimental averages and the simulated bridging bidentate bond distances.

As mentioned in the introduction our HOMO-LUMO (HL) gap for the optimized (TiO_2_)_5_ is 4.45 eV, which is within a few tenths of an eV calculated for much larger nanoclusters. Our optimized nanocluster has C_1_ symmetry and contains three terminal Ti-O bonds with a 1.62 Å bond length and seven bridging O atoms. All Ti atoms in the structure are 4-fold coordinated, which is the same as a (TiO_2_)_5_ nanocluster of C_1_ symmetry [58], which is 0.53 eV less stable than the global minimum. For comparison, the structure with the global minimum has an HL gap of 4.54 eV. When the carboxylate of N3 binds to the surface, it breaks a bridging O atom of the cluster, which creates one additional Ti-O terminal group in complexes A and B. We have calculated the binding energy for a singlet N3 anion reacting with neutral (TiO_2_)_5_ to give a minus one charged singlet species for the two complexes.

The binding energy E (binding) for an N3 monoanion reacting with the (TiO_2_)_5_ NP for complex A or B using the B3LYP functional is given by:

E[binding] = E[(TiO_2_)_5_] + E[N3(k)^−^] − E[complex(i)^−^] (1)

where k = cis or trans deprotonated carboxylate and i = A or B complex, and all energies are optimized values in vacuum with a stationary point found after Raman analysis, which shows zero imaginary frequencies. The same basis set and G16 default grid size were used for all calculations, and values are not corrected for the basis set superposition error. For complex A, the binding energy was calculated for cis and trans species to be 72.23 kcal/mol and 70.47 kcal/mol, whereas for complex B, it was 93.16 kcal/mol and 91.54 kcal/mol, respectively. Thus, the binding energies are about the same for either cis or trans deprotonated N3^−^ species. On the other hand, complex B is about 21 kcal/mol more stable than complex A. However, the normal Raman scattering (NRS) spectrum for complex A is calculated to be twice as intense as that for complex B, so that it would dominate Raman scattering if both occurred. Thus, for correlating the Raman spectrum with structure on titania surfaces, complex A is considered the more important structure.

### 3.3. Electronic Structure of the Complex

We will mostly be concerned with complex A, which we will refer to as N3–(TiO_2_)_5_^−^. This complex has an electronic structure with a set of 13 molecular orbitals that span an energy region, which can be considered to correspond to the HL band gap of the Ti_5_O_10_ NP. These molecular orbitals are listed in Table 2. We note that this complex has been constructed as a singlet anion assuming that the N3 anion with a deprotonated carboxylate binds with the neutral Ti_5_O_10_. Thus, it is interesting that the molecular orbitals of the N3 part of the complex closely resemble those of neutral N3 and not those of the N3 anion. However, this fact is consistent with the high positive charge on the two Ti atoms, which bind the two negatively charged O atoms on the carboxylate anchor of the dye and act similar to the protons in the free N3. Thus, the HOMO and the HOMO-1 to HOMO-7 orbitals match closely the same eight orbitals of free N3 dye [28], which is a fact that has been previously observed for the combined system [30]. The HOMO and the seven MOs below it contain mixed Ru 4d and isothiocyanate orbitals [28,30,59]; see Table 2. Since the Ru-N bond distances are unequal and the octahedral structure is distorted in the complex, similar to the free N3 (Table 1), the Ru 4d orbitals do not show the typical ligand field splitting of a d^6^ ion in an octahedral field.

In fact, the calculated N(bpy_trans_)-Ru- N(bpy_cis_) angle in the complex is 78.9°, which is similar to 78.5° calculated for N3 and to 79.5° for the X-ray structure of N3 (Table 1), showing a strong distortion from 90° of the octahedral structure. In addition, the SCN-Ru-N(bpy_trans_) angle, 173°, in the complex deviates from 180° as it does in N3. These two types of L-M-L distortions of the octahedral geometry split the degeneracy of the t_2g_ set [60], as seen in Table 2.

In fact, not one of the six ligand-donating orbitals is along the z-axis, and they are also located in between the axes. A Natural Bond Orbital, NBO, analysis of N3 shows the natural atomic orbital energies and occupancies of the d orbitals as 4d_x2-y2_, −6.34 eV (1.73); 4d_xz_, −6.07 eV (1.50); 4d_z2_, −5.95 eV (1.53); 4d_yz_, −5.54 eV (1.15); 4d_xy_, −5.49 eV, (1.18). The natural bond orbital analysis shows two strong NBO bonds for the two Ru-NCS bonds with 1.97 occupation and 75% of the electron density on the sp N and 25% on the Ru hybrid. The three lone pair Ru occupations are: LP(1) (1.91), LP(2) (1.84), and LP(3) (1.73). This NBO analysis shows the splitting of the d atomic orbital block and the polar covalent character of the six dative bonds between the ligands and Ru in this 18-electron distorted octahedral system. We have also examined bond orders for these Ru-N bonds. The Wiberg bond indices from the NBO analysis for isolated N3 are 0.625 for two Ru-N (NCS) bonds, 0.544 for two Ru-N (bpy_cis_) bonds, and 0.554 for two Ru-N(bpy_trans_) bonds. In addition, we calculated Mayer bond orders for Ru-N bonds in the N3-(TiO_2_)_5_^−^ complex with Multiwfn software [57]. These are 0.754 for the Ru-N (NCS) bond trans to the anchoring carboxylate group, 0.747 for the other Ru-N (NCS) bond, 0.666 for the Ru-N (bpy_cis_) and 0.672 for Ru-N(bpy_trans_) bonds on the bpy with the carboxylate anchoring group, and 0.722 for Ru-N (bpy_cis_) and 0.733 for Ru-N(bpy_trans_) bonds on the other bpy ligand.

We find in the complex that below the HOMO, there is one set of three quasi-degenerate MOs HOMO-1 to HOMO-3 (H-1 to H-3). These can be represented by the iso-surface of HOMO-1 (Figure 5A), which contains a Ru 4d_yz_ orbital and p orbitals on the S and N atoms of NCS, which are anti-bonding with respect to each other. Below this set of three MOs, there is another set of four quasi-degenerate MOs from HOMO-4 to HOMO-7 (H-4 to H-7); see Table 2. HOMO-4 is unique for both the free N3 and the complex, since it does not contain a Ru orbital but only contains p orbitals on N and S atoms, which are also antibonding with respect to each other [28]. The three very similar molecular orbitals H-5 to H-7 contain a Ru 4 d_Z_^2^ and a π bonding C = S combination and are represented by HOMO-7 (Figure 5B). Here, there is also some leakage around the Ru 4 d_Z_^2^ orbital, which is bonding with the N atom of the NCS group. The HOMO-1, which is shown in Figure 5A, is also very similar to the HOMO and the HOMO-2 and HOMO-3 molecular orbitals.

**Figure 6:**
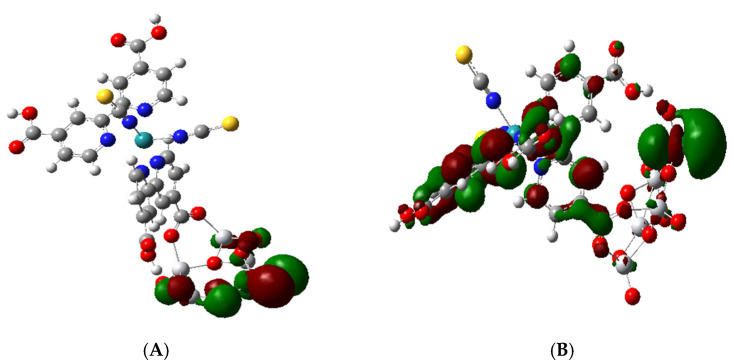
Molecular orbitals representing the band edges. (**A**): HOMO-8 showing atomic p orbitals of O atoms in (TiO_2_)_5_. (**B**): LUMO + 3 showing the 3d_Z_^2^ orbital of Ti atoms of (TiO_2_)_5_.

The atomic orbitals for the TiO_2_ NP first appear in molecular orbital HOMO-8 corresponding to the valence band edge. Thus, the HOMO-8 MO contains only 2p atomic orbitals of the O atoms and 3d_xy_ orbitals of Ti and is a pure MO of the TiO_2_ nanocluster; see Figure 6A. The next four MOs below H-8 going down to H-12 at −6.28 eV are similar to it and thus are all pure TiO_2_ MOs. Starting at HOMO-13, there is a mixed MO iso-surface which contains C = C π bonding in the bipyridine and O p orbitals on the cis COOH group of the bipyridine that binds to the NP as well as 2p orbitals on the O atom of the NP.

**Figure 7:**
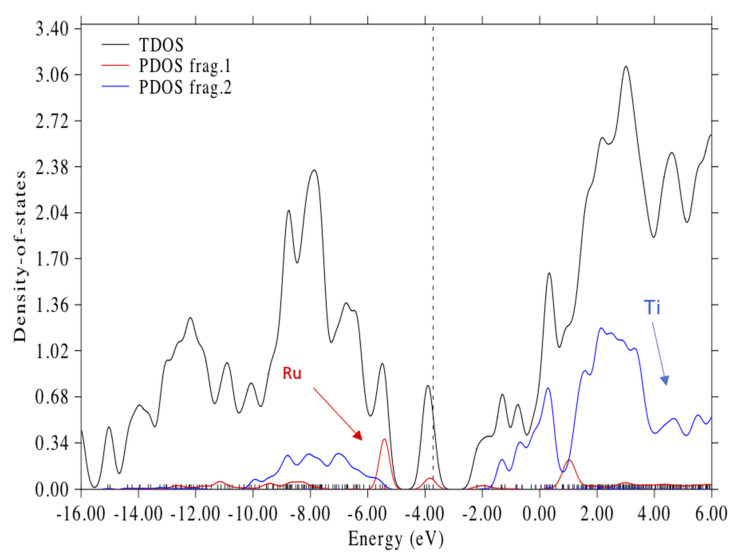
Total density of states (TDOS) and partial density of states (PDOS) for the N3–(TiO_2_)_5_^1−^ complex. Frag. 1 (red) indicates states with the Ru atom. Frag 2 (blue) indicates states with Ti atoms. Gaussian broadening with 0.4 eV FWHM. Dotted line is at the HOMO energy 3.717 eV. Hirshfeld analysis was used for calculating orbital compositions with Multwfn 3.8 software [56].

The LUMO + 3, which represents the conduction band edge, is a mixed MO with anti-bonding π* orbitals in both bipyridine rings and a Ti 3d_Z_^2^ orbital; see Figure 6B. The energy difference between HOMO-8 and LUMO + 3 represents the approximate band gap and spans an energy of 4.39 eV. The total density of states (TDOS) and the partial density of states (PDOS) for Ru and Ti illustrating the putative band gap are shown in Figure 7. The approximate band gap can be seen in the Ti partial density of states plot (blue line). The putative band gap of the complex, 4.39 eV, is close to the 4.45 eV HL gap for the isolated (TiO_2_)_5_ NP. The HOMO at 3.72 eV is 2.02 eV above the putative valance band edge (H-8) and 2.37 eV below the putative conduction band edge (L + 3); see Figure 7. The HOMO, dotted line in Figure 7, is seen to be almost in the middle of the presumed band gap. There are three molecular orbitals above the HOMO and below LUMO + 3 in the putative band gap, which are the LUMO, LUMO + 1, and LUMO + 2, all containing anti-bonding π * C = C combinations of the bipyridine rings and unoccupied Ru 4d orbitals; see Table 2 and Figure 7. The LUMO + 2 is 0.3 eV below the “conduction band edge” MO (L + 3). These MO energy levels are similar to the calculations of Persson and Lundqvist [30], where the penultimate MO in the band gap was 0.2 eV below the conduction band edge in the much larger (TiO_2_)_38_ NP. Thus, the ground state electronic structure in our model is similar to the N3-(TiO_2_)_38_ model [30] except for the fact that our MO energies are shifted up ca. 1.5 eV because of the negative charge on the complex.

In the N3-sensitized DSSCs, fast electron injection has been thought to involve MLCT transitions in a two-step process where the excited states of N3 are well above the conduction band of TiO_2_ films [42]. However, it is the excitation energies and not the ground state unoccupied energy levels that are important for the photoinduced CT process. We discuss these excited states in the next section. Our N3–(TiO_2_)_5_^−^ model complex appears to be similar to the electronic structure of the N3–(TiO_2_)_38_ model, and thus, it should be adequate to investigate optical absorption and Raman scattering spectra for a surface site with the bridging bidentate structure.

### 3.4. Simulated Optical Absorption Spectra

**Figure 8:**
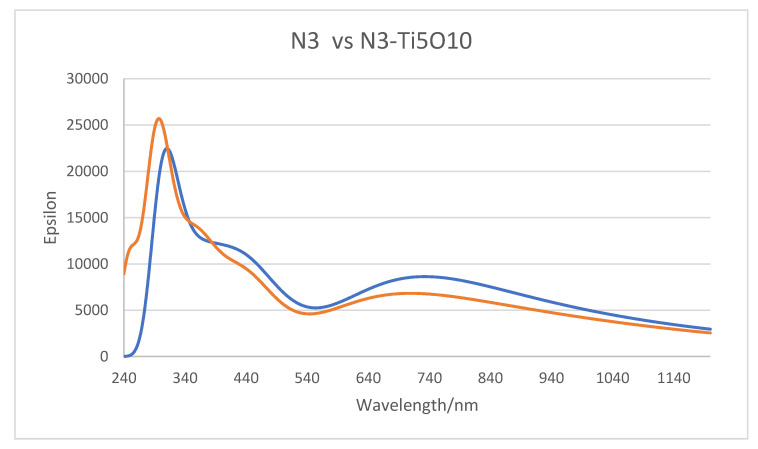
Absorption spectra of N3 dye (red) and the N3–(TiO_2_)_5_^−^ complex (blue) simulated in vacuum. Full width at half maximum, FWHM broadening is 0.300 eV.

In Figure 8, we have plotted the TD-DFT simulated optical absorption curves for N3 and the complex A, N3-(TiO_2_)_5_^−^, which are both obtained in vacuum. The two spectra are for the most part very similar. One difference in the two curves is that the shoulder at 250 nm for N3 is not reproduced for the complex. This is an artefact stemming from that the fact that even with 150 excited states, the B3LYP/6-31 + G(d)/LANL2DZ simulated spectrum does not quite go down to 240 nm where the N3 spectrum starts. This calculation involved 2081 primitive Gaussians contracted to 1006 symmetry adapted basis functions. The other more important difference is that the spectrum (blue) of the complex is slightly red shifted from the N3 spectrum, which is due to a number of very weak charge transfer excitations between the dye and the nanocluster.

The above result is akin to experimental results where nearly identical absorption curves were found for N3 in DMSO and for colloidal N3|TiO_2_ in DMSO [23]. In addition, similar curves for N3 in ethanol solution and N3 adsorbed on nanocrystalline TiO_2_ films were observed [21]. This observation that the low-energy bands of N3 and N719 in solution and on TiO_2_ are very similar has been attributed to the similar roles that Ti^4+^ in the adsorbed state and protons in solution play in binding the oxygen atoms of carboxylate [21]. This conclusion is supported by our calculations, which show that the binding Ti atoms in our NP have a very high positive charge and are also the reason why the simulated N3 and N3–(TiO_2_)_5_^−^ absorption spectra are so similar.

**Figure 9:**
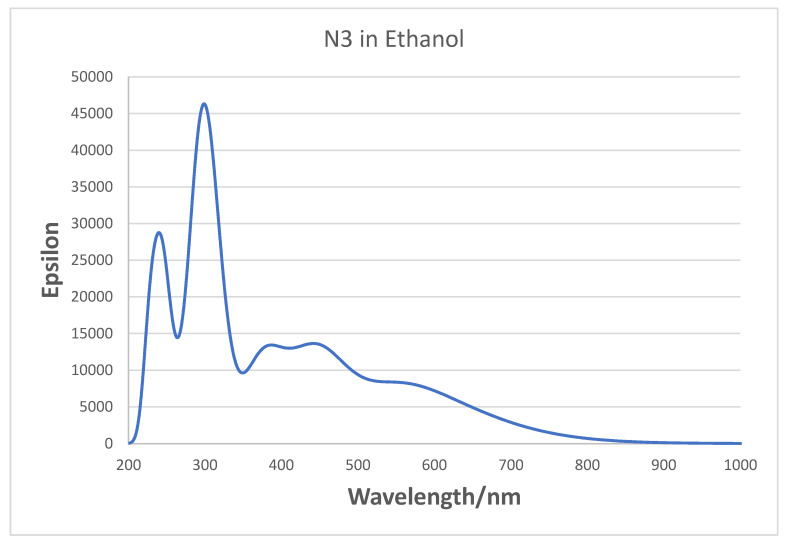
Absorption spectra of N3 dye simulated in ethanol using IEFPCM. FWHM broadening is 0.25 eV.

For N3 in ethanol solution, the MLCT bands in the experimental spectra were assigned at 398 nm and 538 nm in the visible, and bands at 314 nm and a shoulder at 304 nm in the UV were assigned to intra ligand π–π * transitions [21,60]. In our gas phase UV-VIS simulation of neutral N3 in Figure 8, we observe bands around 300, 370, 440, and 700 nm, which are quite different from the experimental spectrum. The largest difference is that the experimental peak at 538 nm appears to be shifted to 700 nm in the simulation. The TD-DFT calculations show this latter band is an MLCT excitation. Our three highest wavelength bands for N3 are very similar to those found by Kouki et al. in vacuum [57] at 373, 446, and 670 nm using B3LYP and a much larger basis set. In aqueous solution, the pH effect should be considered, which determines the charge on N3. At pH 11, the peaks are found at 308, 370, and 500 nm [5] presumably from the N3 dianion. We have also examined the effect on the gas phase absorption spectrum of deprotonation of N3. In Figure S1, the neutral N3, its singly charged anion, and the dianion (same as the dianion of N719) are compared. The peak for N3 at 300 nm is shifted to 320 nm in the N3 anion and 340 nm in the dianion; however, the peak in the visible at around 700 nm does not shift very much or vary systematically with charge. On the other hand, it is well known that theoretical simulations of Ru dyes, which include a solvent environment, give a much better representation of the experimental spectra [58,60,61]. In fact, if we simulate the spectrum of N3 in ethanol, as seen in Figure 9, we do find bands at 300, 378, 458, and 561 nm in the visible, which are much closer to experimental values. However, we could not use a solvent environment in our Raman simulations with the N3–(TiO_2_)_5_^−^ complex, because in this case, the calculations would not converge. Therefore, we were forced to conduct the vibrational calculations in vacuum. Nevertheless, it should be noted that Raman simulations in vacuum show excellent agreement with experimental vibrational Raman frequencies in solution after an empirical scale factor correction. Thus, we use the TD-DFT vacuum excitation calculations to analyze the nature of the excited states that are used for exciting time-dependent Raman spectra and to analyze the CT process.

### 3.5. Excited State Transitions

Here, we report on the TD-DFT calculations of the optical transition of N3–(TiO_2_)_5_^−^ in vacuum corresponding to the spectrum of the complex in Figure 8. We calculated 150 excited states of the complex; however, we will only be concerned with the first 45 excited states, which cover the near IR to around 400 nm. It turns out that the first 13 excited states cover the spectral region of 1200 to 648 nm and are all very similar to previous calculations for N3 [28]. These excitations have been described as MLCT transitions from mixed Ru isothiocyanate orbitals to the low-lying π* orbitals of the dcbpy ligands. For our complex, the transitions described in the TD-DFT simulations all involve combinations of many delocalized molecular orbitals in both the hole and particle/electron parts of the excitations. Thus, in the first 10 excitations, all the hole states are composed of various combinations of HOMO, HOMO-1, HOMO-2, and HOMO-3, while the particle states involve various combinations of the LUMO, LUMO + 1, and LUMO + 2. These are in the gap below the putative conduction band edge; see Figure 7. We illustrate these transitions for representative excitations with the natural transition orbital, NTO, method of Martin [53] for hole and particle states. NTOs utilizes an orbital transformation that brings the transition density to diagonal form and allows a dramatic simplification in the qualitative description of electronic transitions.

**Figure 10:**
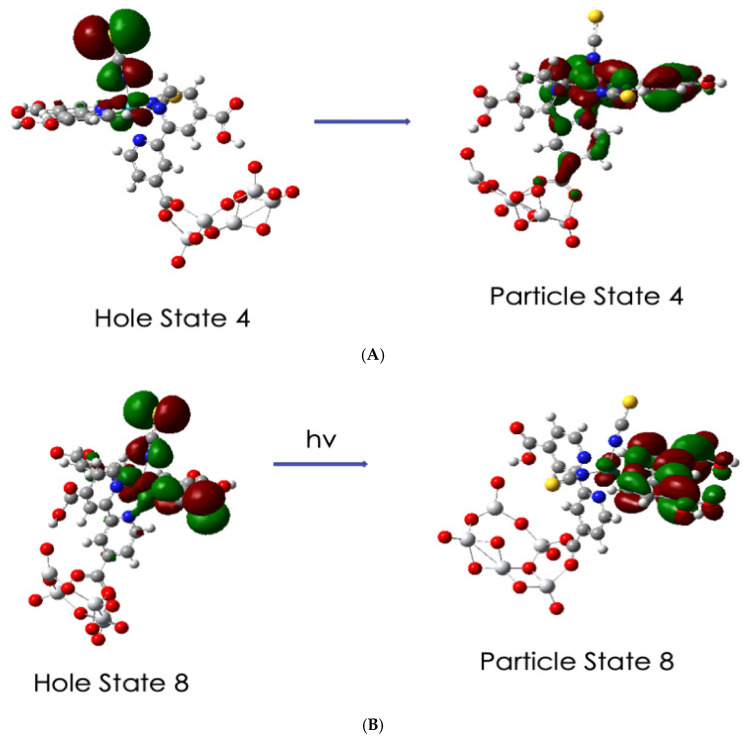
Hole and particle natural transition orbitals for excited states 4 and 8. (**A**). Excited state 4, energy = 1.2770 eV, *f* = 0.0151. (**B**). Excited state 8, energy = 1.6379 eV, *f* = 0.0624.

The simplification is observed in Figure 10A where excited state 4 at 1.28 eV with oscillator strength *f* = 0.0151 is represented by five MO transitions, as shown in Table S1, with various coefficients in the TD-DFT output but by easily recognized orbitals in the hole/particle NTO iso-surfaces. This transition includes the MLCT of Ru-NCS hole to a particle with π* dcbpy, which is not anchored and also to the anchored π* pyridine and carboxylate part of the complex. In addition, it includes a ligand field transition from Ru 6d_yz_ to Ru 6d_z_^2^. The excitation for excited state 8, Figure 10B, at 1.64 eV and *f*= 0.0624 is a stronger excitation, which involves seven MO transitions. Its hole/particle NTOs involves the transition of the Ru–(NCS)_2_ hole to a particle with the non-anchored π * dcbpy. However, it also includes a ligand field transition of Ru 6d_z_^2^ to Ru 6d_yz_. Both transitions in Figure 10 are below the energy of the LUMO + 3, which represents the conduction band edge and would not involve charge transfer to the TiO_2_ part of the complex; however, they would support resonance Raman excitation.

**Figure 11:**
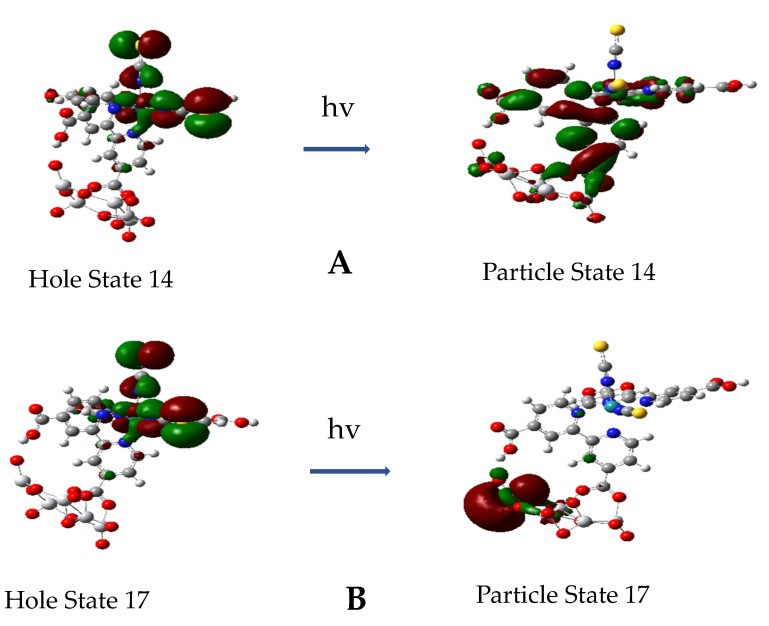
Hole and particle natural transition orbitals for excited states 14 and 17. (**A**): State 14, energy = 2.0049 eV, f = 0.0314. (**B**): State 17, energy = 2.1275 eV, f = 0.0009.

All excitations for the first 45 excited states for the complex up to around 3.2 eV or 392 nm involve transitions from a hole state, which is either a Ru-NCS or Ru-(NCS)_2_ NTO, and these are both illustrated in Figure 10. The first thirteen of these excited states are mainly excitations to π* parts of the dcbpy ligands with some involvement of d orbitals of Ru. Starting at excited state 14, the excitation is a mixed transition with the particle state involving π* dcbpy ligands, including the anchoring carboxylate and some 3d_Z_^2^ orbitals on the Ti atoms; see Figure 11A. This excitation is at 2.0 eV with *f* = 0.0314. However, at excited state 17, the transition becomes a pure direct photoinduced charge transfer from Ru-NCS to the Ti 3d_Z_^2^ orbital; see Figure 11B. This is similar to the direct photo-injection mechanism in the calculations of Persson et al. [35] for catechol on a Ti_38_O_76_ nanoparticle model. Furthermore, excited state 17 is more akin to excited state 18 at 1.98 eV of N719-(TiO_2_)_82_ complex of De Angelis et al. [33], which shows an electron state dominated by Ti(iV) t_2g_ orbitals with *f* = 0.03. For our model, excited state 17 at 2.13 eV has an oscillator strength of only *f* = 0.009, but it should support Herzberg–Teller scattering in a CT-SERS mechanism. These results indicate the possibility of a direct one-step CT mechanism for DSSCs.

The excited states were also examined with the charge transfer distance index, D_CT_, which measures the spatial extent of charge transfer excitations in Å and the charge passed index, q_CT_, which is the integration of the density depletion function over all space in atomic units, au. The index D_CT_ is calculated as the distance between the barycenters of density increment and density depletion regions. Table S2 shows the first hundred excited states with their values of D_CT_, q_CT_, and oscillator strengths. We arbitrarily assume that any state with a charge transfer distance index over 9.0 Å and charged passed index over 0.980 au is a charge transfer state. There are 10 such states in the first hundred excited states of the complex in Table S2, which have nonzero oscillator strength. For example, state 17 shown in Figure 11B has values of D_CT_ = 10.426 Å, q_CT_ = 0.985 au with an oscillator strength of 0.0009. State 21 which has hole/particle NTO iso-surfaces identical to those of excited state 17 has values of D_CT_ = 10.099 Å, q_CT_ = 0.982au with an oscillator strength of 0.0003. These 10 long-distance CT states have an oscillator strength average of only 0.0003, indicating little overlap between the ground and excited state wavefunctions; however, they all could support the CT-SERS mechanism by intensity borrowing.

### 3.6. Simulated Raman Scattering Spectra

**Figure 12:**
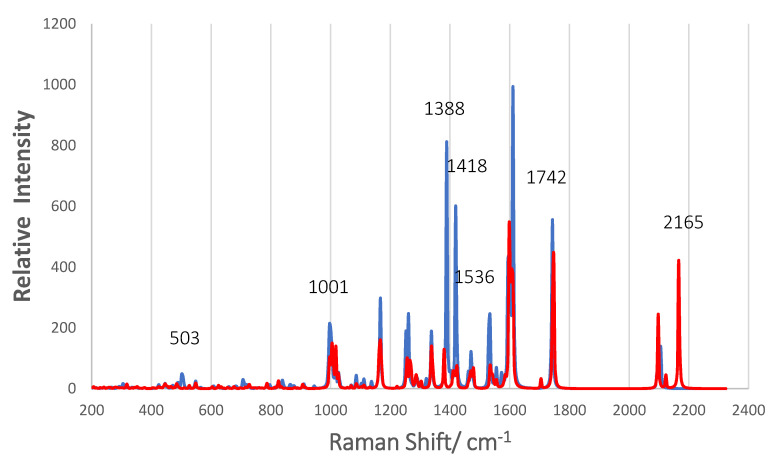
Normal Raman spectra of complex A (blue) and complex B (red). The intensity of complex B has been doubled for clarity. FWHM broadening 2 cm^−1^. A scaling factor 0.970 have been used for all Raman frequencies.

Figure 12 shows the normal Raman scattering (NRS) spectra of the two complexes A and B. Both are optimized structures of N3 anchored by a bridging bidentate carboxylate on Ti_5_O_10_, as shown in Figure 4, but complex B is tilted so that one of its isothiocyanate ligands is within 2.5 Å of the nanoparticle surface. The intensity of the NRS spectrum of complex B was doubled for clarity in Figure 12. For comparison, the strongest peak in both complexes at 1610 cm^−1^ is actually 2.3 more intense in complex A. The most striking feature of complex B is the two peaks around 2100 cm^−1^. The stronger peak at 2165 cm^−1^ is the C = N vibration of the NCS ligand closest to the surface, while the peak at 2097 cm^−1^ is for the NCS ligand away from the surface. Only one peak at 2104 cm^−1^ shows up for complex A, which is the C = N vibration for both NCS ligands. There is also a very small peak for complex B in this region at 2122 cm^−1^ in between the more intense ones, which is the O-H vibration for –COOH forming a hydrogen bond to an O atom of Ti_5_O_10_. The effect of the surface on the C = N stretch is to split the vibration into two and to enhance the one for the NCS ligand closest to the surface. The band at 2165 cm^−1^ is close to the strong band at 2156 cm^−1^ for the pre-resonance Raman of N719 excited with 632.8 on silver colloid in acetonitrile where strong adsorption via the NCS groups was proposed [13]. A Ag_2_@TiO_2_ dimeric-shell–core NP also shows the band at 2155 cm^−1^ for N719, again indicating it is adsorbed via the two NCS groups [16]. This peak for N719 moves to 2104 cm^−1^ on TiO_2_ surfaces [5,22,23] as we find with our simulation of complex A in which the two NCS groups are oriented away from the NP surface. As far as we know, experimental Raman spectra on TiO_2_ surfaces have not shown two bands around 2100 cm^−1^, which indicated that this tilted surface geometry of complex B is not prevalent.

A comparison of the NRS for complex A and B shows that in the frequency range of 1000 to 1800 cm^−1^, most major bands overlap with exceptions of a few weak bands such as the one at 1709 cm^−1^. In Table 3, we compare our calculated NRS of complex A with four reports of experimental spectra in the literature. The experimental results are for N3 or N719 on various TiO_2_ surfaces and in different solvents with reported excitation at 780, 514.5, 514, and at 415.5 nm. Considering the diversity of the experiments, the agreement of the Raman shift frequencies between the experiments and with our simulation is very good. This is reasonable, since bound N719 and N3 are alike because Ti(IV) supply positive charge to the anchoring groups, and the lack of symmetry shows the same frequencies even though excitation at 780 nm is NRS and 514 nm and 415 nm would give RRS. Important results revealed by the normal mode analysis of the N3-(TiO_2_)_5_ complex is that the band at 1534 cm^−1^ is not just a bipyridine ring stretch but contains O = C = O stretching of the anchoring group, which is in phase with the C = C stretching in the pyridine to which it is attached (see Figure S5). In addition, the band at 1388.5 cm^−1^ is shown to have the assignment of the symmetrical COO stretch for the carboxylate anchoring group on (TiO_2_)_5_ in the bridging bidentate structure, which is in-phase with C–H wags on its pyridine ring; see Figure S4. The assumption made in the literature has been that the band at 1388 cm^−1^ indicates either the bridging bidentate or the bidentate chelate binding [23,24]. Our result is evidence that this band is for the bridging bidentate structure. Shoute and Loppnow [23] indicate that this band was not observed in the Raman spectrum of isolated N3, and our simulation of the NRS spectrum of isolated N3 allows the same conclusion (Figure S2). Thus, it appears that the band at 1388 cm^−1^ indicates chemisorbed N3 (N719) in a bridging bidentate structure. In fact, there are three vibrational modes at 503, 1388, and 1534 cm^−1^ that contain vibrations of the anchoring carboxylate group. The displacement vectors of these normal modes are shown in Figures S3–S5. Furthermore, some of the low-frequency modes in Table 3 contain vibrations of the (TiO_2_)_5_ NP such as 836 and 796 cm^−1^.

Finally, we have simulated with TD-DFT the frequency-dependent Raman scattering spectra by exciting near two different excited states. These two excited states are S4 and S17, and their hole/particle NTOs are shown in Figure 10A and Figure 11B, and their MO compositions are given in Table S1. Excited state S4 is an MLCT excitation at 1.277 eV (*f* = 0.0151), which is below the putative conduction band edge and should give resonance Raman scattering by a Franck–Condon scattering mechanism. Excited state S17 at 2.1275 eV (*f* = 0.009) is long distance charge transfer excitation (D_CT_ = 10.43 Å) right at the putative conduction band edge and should give Raman scattering by intensity borrowing from a nearby strong state through a Herzberg–Teller scattering mechanism [9].

The TD-DFT simulations are dynamic polarizability calculations that follow the outline of Neugebauer et al. [62] and are based on the Placzek theory with the double harmonic approximation (harmonic oscillator force field and truncation of the Taylor expansion of the polarizability with respect to a normal mode after the quadratic term). This requires numerical derivatives of the polarizability tensor components with respect to a normal coordinate. The dynamic polarizability is found at each band position by calculating the frequency-dependent polarizability using pre-resonance Raman code [50]. If we formulate the dynamic polarizability tensor components in terms of the Kramers–Heisenberg–Dirac (KDH) expression through second-order perturbation theory [63], using the Born–Oppenheimer approximation, expanding the electronic transition moments, *μ*, in a Taylor expansion to first power in the normal coordinates Q, and utilizing the Herzberg–Teller, HT, formulation for mixing of vibronic functions, we obtain Equations (2)–(4)with the following familiar A and B terms of Albrecht [64] as we developed [63]

(2) $$\alpha_{\sigma \rho }=A+B$$

(3) $$A=\sum_{S=F,K\neq I}\sum_{k}\left[\frac{\mu_{SI}^{\sigma }\mu_{SI}^{\rho }}{\hbar \left(\omega_{SI}-\omega \right)}+\frac{\mu_{SI}^{\rho }\mu_{SI}^{\varrho }}{\hbar \left(\omega_{SI}+\omega \right)}\right]\langle i\left|k><k\right|f\rangle ,$$

(4) $$\begin{array}{cc} B= & \underset{R=F,K}{\sum }\underset{S=F,K}{\sum }\left[\frac{\mu_{IR}^{\sigma }h_{RS}\mu_{SI}^{\rho }}{\hbar \left(\omega_{RI}-\omega \right)}+\frac{\mu_{IR}^{\rho }h_{RS}\mu_{SI}^{\rho \varrho }}{\hbar \left(\omega_{RI}+\omega \right)}\right]\frac{\langle i\left|k>\langle k|Q_{k}\right|f\rangle }{\hbar \omega_{RS}}+\\ & \;\underset{R=F,K}{\sum }\underset{S=F,K}{\sum }\underset{k}{\sum }\left[\frac{\mu_{IS}^{\sigma }h_{SR}\mu_{RI}^{\rho }}{\hbar \left(\omega_{RI}-\omega \right)}+\frac{\mu_{IR}^{\rho }h_{RS}\mu_{SI}^{\rho \varrho }}{\hbar \left(\omega_{RI}+\omega \right)}\right]\frac{\langle i\left|Q_{k}\right|k\rangle <k|f\rangle }{\hbar \omega_{RS}} \end{array}$$

where *E_SI_* = $\hbar \omega_{SI}$ = *E_S_ − E_I_*, the energy between the ground electronic state I and an excited electronic state S, etc., the exciting light is *h*ω, <*i*|, and |*f* > are initial and final vibrational wavefunctions of the ground state, <*k*| is the wavefunction of an intermediate state, and *σ*, *ρ* are the polarization directions. When there is a resonance, the second term in the square brackets of each summation is negligible with respect to the first term. In these equations, we have not included the imaginary bandwidth, *iγ_k_*, in the resonance denominator. The A-term is the Franck–Condon term responsible for molecular resonance Raman, and the B-term is Herzberg–Teller term responsible for a long-range charge-transfer resonance between molecule and nanoparticle, CT-SERS. In these expressions, the sums range over all excited states (*R* and *S*), which include both charge transfer states and molecule or nanoparticle states (but of course exclude terms for which a denominator vanishes (such as *S* or *R* = *I*)). In order to examine the situation in which the light is not only resonant with the molecular excitations but also a charge-transfer transition in the nanoparticle–molecule system, we select for examination only terms in the general expression (1) above that include a charge-transfer state. These terms contain the linear Herzberg–Teller vibronic coupling constant $h_{SR}$

(5) $$h_{SR}=\langle S_{e},\;0\left|{\left(\frac{\partial V_{eN}}{\partial Q}\right)}_{0}\right|R_{e},0\rangle \;$$

where *V_eN_* is the electron–nuclear attraction term in the Hamiltonian, which is evaluated at the equilibrium nuclear positions (0), and the purely electronic transition moment between states are written: *μ^σ^_SI_* = <*S_e_*|*μ^σ^*|*I_e_*>, *μ^σ^_RI_* = <*R_e_*|*μ^σ^*|*I_e_*> and *μ^σ^_SR_* = <*S_e_*|*μ^σ^*|*R_e_*>. Since in long-range charge transfer, the dipole moment μ_SI_ of Equation (2) is very small, it is the B term in Equation (3) above that can dominate the A term and give rise to charge transfer Raman scattering when there is coupling between electronic excited states R and S with finite Herzberg–Teller vibronic coupling constant [65].

**Figure 13:**
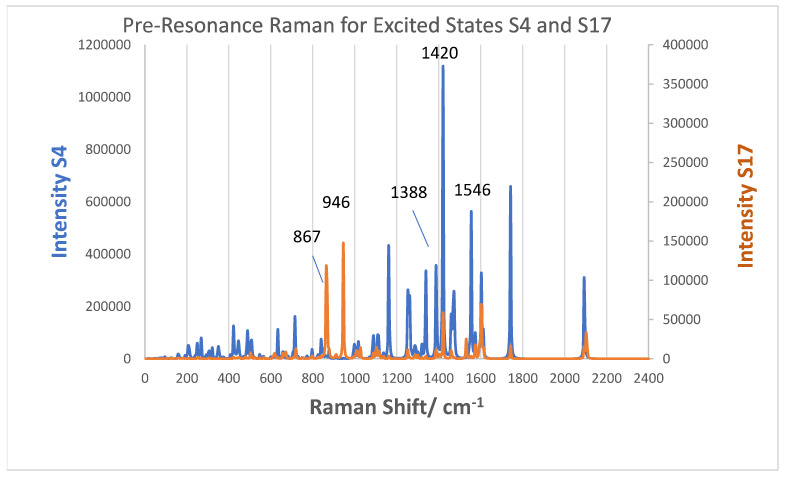
Pre-resonance Raman spectra of N3–(TiO_2_)_5_ for complex A. Blue spectrum excited near S4, *f* = 0.0151 at 955.27 nm (10,468.9 cm^−1^). Orange spectrum excited near state S17, *f* = 0.0009 at 587.20 nm (17,029.9 cm^−1^) FWHM broadening 2 cm^−1^. A scaling factor 0.970 has been used for the Raman frequencies.

The simulated pre-resonance Raman spectra for excitation near excited state 4, which is a MLCT transition, and near excited state 17, which is long-distance dye-to-TiO_2_ CT transition, are illustrated in Figure 13. The excitation for the former spectrum (blue) at 955.3 nm is 169 cm^−1^ above excited state 4, while the excitation for the latter spectrum (orange) is 130 cm^−1^ below excited state 17. The vertical axis for state 4, the MLCT transition, is on the left of Figure 13 and is a factor of three higher than the vertical axis for state 17, the N3-to-(TiO_2_)_5_ CT transition, on the right of the figure. The MLCT pre-resonance Raman spectrum is about 6.5 times more intense than the N3-to-(TiO_2_)_5_ CT pre-resonance Raman spectrum. On the other hand, the ratio of oscillator strength is about 17 higher for the MLCT process compared to the N3-to-TiO_2_ CT process. In fact, the pre-resonance spectrum for excited state 17 and for excited state 4 are about 2 × 10^2^ and about 1.3 × 10^3^ times the simulated normal Raman spectrum, respectively. The fact that the oscillator strength for excited state 17 is so low and yet there is significant enhancement of the pre-resonance Raman spectrum indicates the intensity borrowing mechanism of Equation (3) is operating.

There are some bands that become more intense in these pre-resonance Raman spectra. The two strongest bands in the N3-to-(TiO_2_)_5_ CT spectrum are at 867 and 946 cm^−1^, and both involve the (TiO_2_)_5_ NP. They are a Ti–O stretching vibration coupled to the O–H bend of the carboxylate group hydrogen bonded to the vibrating Ti–O group (946 cm^−1^) and an out-of-plane C–H wag in the pyridine-ring whose carboxylate is H-bonded to the Ti–O (867 cm^−1^). These C–H wags are also coupled to the Ti–O–Ti stretching vibration in the nanoparticle. The group of bands in the MLCT pre-resonance Raman spectrum at 1611, 1573, and 1472 cm^−1^ have the same intensity ratios as experimental MLCT resonance Raman spectra of N3 or N719 on titania in the literature [5,23,25]. In addition, the 1388 cm^−1^ for the symmetrical COO stretching vibration is still observable in both pre-resonance spectra. A noticeable difference in the MLCT RRS is the band at 1420 cm^−1^, which becomes the most intense band in the spectrum. This vibration (Figure S6) contains in-plane C–H wags on all the bipyridine ligands but also has a contribution from the symmetrical O = C=O stretch of the anchoring carboxylate group. It is somewhat similar to the normal mode at 1388.5 cm^−1^. In fact, there is a weak band in the resonance Raman spectrum excited at 414.44 nm of Nazeeruddin et al. [5] at 1430 cm^−1^ and a band reported at 1433 cm^−1^ reported by Greijer at al. [22] which may correspond to this band. Since the excited states of the N3–(TiO_2_)_5_^1−^ complex, which is an anion and a very small model NP system, will not correspond to those of N3 or N719 adsorbed on a much larger TiO_2_ surface, which should have a continuum of conduction band states, it is difficult to correlate band intensities of these simulated pre-resonance spectra with experimental frequency dependent spectra. However, the normal modes that we have determined with the simulation of these spectra should certainly be valid and of interest for future experimental studies.

## 4. Conclusions

The geometrical structure of the Ruthenium dicarboxylbipyridyl dye N3 (whether in its isolated neutral and anionic forms or as an adsorbate bound to a (TiO_2_)_5_ NP) was found by DFT calculations to have the same distorted octahedral structure, which splits the d orbital manifold of Ru. The bound complex had two configurations, both which anchor N3 through a carboxylate bridging bidentate geometry to two Ti atoms and also involve a hydrogen bond formed by the other –COOH group on the binding bipyridine to a dangling O atom of the NP. One form of the complex has the isothiocyanate groups directed away from the surface, while a more tightly bound form, as indicated by binding energy calculations, has only one of the isothiocyanate groups interacting with the surface. This form of the surface geometry can be detected, since it has two N = C stretching vibrations from the NCS groups at 2104 and 2165 cm^−1^, whereas the other form with NCS groups directed away from the surface shows only the 2104 cm^−1^ stretching vibration. The strong interaction of NCS groups from N3 or N719 is more likely to be observed on metals like Au or Hg and has been detected on the Hg and Au [12,13,17,18] and on Ag@TiO_2_ and Ag_2_@TiO_2_ [15,16], where in all these cases, only the higher wavenumber vibration shows up in the spectra, indicating both NCS groups binding strongly to the surface.

The simulations also show that the band at 1388 cm^−1^, which contains the symmetrical stretching vibration of the bridging bidentate carboxylate group and which does not show up in the isolated dye, clearly indicates chemisorption of the bound dye with this surface geometry, as deduced experimentally from bands close to 1380 cm^−1^ [18,19,23]. For most of the bands, the DFT calculations show that the normal Raman spectrum of N3 adsorbed on TiO_2_ is similar to isolated N3 because of the positive charge on the Ti atoms. These form a chemical bond with the two oxygen atoms of the carboxylate of the dye similar to hydrogen ions in solution. This conclusion was previously deduced from an experimental optical absorption investigation in the literature [21]. Thus, our DFT structure and Raman calculations confirm many of the explanations inferred from experimental studies. On the other hand, the simulated pre-resonance spectra of both types of resonance mechanisms show enhancement of vibrational modes involving combined dye and TiO_2_ vibrations, such as those at 867, 946, and 1420 cm^−1^.

The electronic structure calculations made for N3–(TiO_2_)_5_ agree with similar calculations made for much larger dye-(TiO_2_)_n_ models (n = 38 or 82) with respect to molecular orbital iso-surfaces and putative band edge gaps. Furthermore, the optimized structural results agree with experimental crystal X-ray average structure data, and thus, N3-(TiO_2_)_5_^1−^ proved to be an adequate model for a single bridging bidentate surface geometry with an additional hydrogen-bonded carboxylate to the NP surface. This relatively small structural model allows time-dependent TD-DFT Raman simulations with a B3LYP/6-31 + G(d)/LANL2DZ calculational model and shows that both two-step intermediate and one-step direct charge transfer mechanisms are possible with N3 dye on TiO_2_. The long-distance dye-to-NP CT is readily determined to be appropriate for all excited states with charge transfer distance index, D_CT_, over 9.0 Å and a charged passed index, q_CT_, over 0.980 au. The optimized geometry with the anchoring group in our model shows Ti-O bond distances of 2.02 Å and 2.06 Å in the C-O-Ti arms of the bridge, indicating a reasonably strong chemical bond anchoring N3 to the surface. The molecular orbital analysis of the N3-(TiO_2_)_5_ complex such as the LUMO + 3 level (Figure 6B) shows delocalization across the anchoring bridge, indicating strong electron coupling facilitating ultrafast electron injection. The Herzberg–Teller resonance Raman scattering theory for long-distance photoinduced CT has implications for photoinduced electron injection at dye-covered TiO_2_ surfaces. Vibronic coupling CT theory analogous to Herzberg–Teller theory has been used to analyze charge-transfer states in organic solar cells [66]. This theory adopts a three-state model involving a ground state photoexcited to a donor-acceptor state, D^+^A^−^, which is vibronically coupled to a local, strongly absorbing excited state on the donor or acceptor.

The dynamic polarizability expression shows that both Franck–Condon and Herzberg–Teller resonance Raman scattering mechanisms are possible depending on transition dipole moments and their derivatives. The MLCT and long-distance dye-to-NP charge-transfer (CT-SERS) pre-resonance Raman scattering mechanisms for the two excited states investigated result in enhancement factors of around 1 × 10^3^ and 2 × 10^2^, respectively. These simulated pre-resonance MLCT and CT-SERS enhancement factors predict many orders of magnitude lower in intensity compared with SERS experiments on Ag, Au, and Ag core–(TiO_2_) shell SERS substrates. However, the enhancement factors are representative for these two types of mechanisms on TiO_2_ surfaces, and the Raman intensities in an experimental case will depend on the oscillator strength of excited states and excited life-time broadening mechanisms. In fact, the optical simulations show many MLCT excited states with two to four times the oscillator strength of the representative excited state 4. Our results suggest that experimental Raman spectra excited at different excitation energies should be able to distinguish between these types of mechanisms on titania surfaces. However, it would be important to extend these studies in future simulations of dye–TiO_2_ Raman spectra to more accurate models. These extensions should include a model with N3 or other dyes adsorbed on a TiO_2_ structure, which allows a quasi-continuum of TiO_2_ conduction band states, implementation of a solvent environment, and modelling of the effect of surface protonation on surface geometry and thus on Raman spectra.

Simple Summary: This study verifies that a small sized model of the semiconductor TiO_2_ can be used to accurately study the properties of Ru bipyridyl dye, called N3, when the dye is adsorbed on its surface. This system is a model of a photoelectrochemical device for conversion of solar energy to electric current called a Dye-Sensitized Solar Cell (DSSC). Various types of Raman spectra were simulated which allow the surface geometry of the dye to be predicted. Also, the mechanisms of how the light photon causes an electron to be injected from the dye into semiconductor surface were investigated by quantum mechanical electronic structure calculations.

## Figures and Tables

**Table 1:** Bond distances in Å and bond angles for our calculated N3 structures compared with two others in the literature and data from an X-ray crystal structure of N3.

Parameters	Calc. B3LYP Vac.	Calc. B3LYP EtOH	Literature [58] B3LYP Vac.	Literature [28] BPW91 Vac. C_2_	Experiment X-ray [27]
Ru-NCS					
Ru-NCS	2.058	2.086	2.045	2.036	2.048
Ru-NCS	2.058	2.086	2.045	2.036	2.046
Ru-N(bpy_trans_)A	2.085	2.086	2.077	2.079	2.036
Ru-N(bpy_trans_)B	2.085	2.086	2.076	2.079	2.058
Ru-N(bpy_cis_)A	2.080	2.094	2.074	2.056	2.030
Ru-N(bpy_cis_)B	2.081	2.094	2.074	2.056	2.013
N = C(NCS)	1.185	1.179	1.178		1.162–1.103
C-S (NCS)	1.628	1.647	1.626		1.615–1.685
<SCN-Ru-NSC	92.6	90.5		90.2	88.7(5)
<N(bpy_trans_)-Ru- N(bpy_cis_)	78.5	78.4		78.9	79.5(5)
<N(bpy_cis_)-Ru- N(bpy_cis_)	177.9	175.2		169.5	174.5(5)
<SCN-Ru-N(bpy_rans_)	172.7	173.8			
<N(bpy_trans_)-Ru-N(bpy_trans_)	93.9	92.6		95.1	90.6(5) 90.6(5)

**Table 2:** Molecular orbitals in the putative band gap of the complex N3-(TiO_2_)_5_^−^.

MO	Energy (eV)	Character
LUMO + 3	−1.34	Ti 3d_Z_^2^ and π* dcbpy on both dcbpy
LUMO + 2	−1.67	π* dcbpy not bound to TiO_2_ and Ru 4d_xz_
LUMO + 1	−1.92	π* dcbpy bound to TiO_2_ and Ru 4d_Z_^2^
LUMO	−2.19	π* dcbpy not bound, Ru 4d_Z_^2^, and on bind. π* pyr-COO
HOMO	−3.72	S 3p_x_, N 2p_x_ on both NCS_,_ Ru 4d_yz_
HOMO-1	−3.86	S 3p_x_, N 2p_x_ on both NCS_,_ Ru 4d_yz_
HOMO-2	−3.94	S 3p_y_, N 2p_y_ on both NCS_,_ Ru 4d_yz_
HOMO-3	−4.02	S 3p_y_, N 2p_y_ on one NCS_,_ Ru 4d_yz_
HOMO-4	−5.36	S 3p_z_, N 2p_z_
HOMO-5	−5.41	C = S π on both NCS, Ru 4d_Z_^2^
HOMO-6	−5.49	C = S π on both NCS, Ru 4d_Z_^2^
HOMO-7	−5.57	C = S π on both NCS, Ru 4d_Z_^2^
HOMO-8	−5.73	O 2p, Ti 3d_xy_

**Table 3:** Comparison of our NRS results for complex A with the literature for N3 or N719 on a TiO_2_ surface. A. TiO_2_/N719 in 3MPN, 780 nm laser [22]. B. N3|TiO_2_ in DMSO, 514.5 nm laser [23]. C. N719-Aqueous TiO_2_, 514 nm laser [25] D. N719 adsorbed on TiO_2_, 415.44 nm laser [5].

A	B	C	D	This Paper	Assignments
2104	2104	2095	2105	2104	(N = C) stretch in NCS
1735		1727	1726	1742	(C = O) stretch in COOH
1611	1610	1605	1610	1613	bpy ring stretch in anchor dcbyp
1544	1541	1542	1539	1534	bpy ring stretch and anchor. O = C=O stretch
	1469	1474	1471	1471	ring stretch in both bpy ligands
1433			1430	1419	i.p. C–H wag on both bpy, C–CO stretch
	1388	1376	1367	1388	i.p. C–H wag on anchored bpy, sym. stretch of anchored COO
1303		1315	1331	1338	i.p. C-H wag on unbound dcbpy, O-H bend on both COOH of dcbpy
1290	1294			1288	i.p. C-H wag on unbound dcbpy, O-H bend on both COOH of this dcbpy
1260	1256	1268	1260	1261	unsym. ring stretch on unbound dcbpy, O-H bend on one COOH
			1222	1252	i.p. C–H wag on unbound dcbpy
	1130		1155	1167	i.p. C–H wag on unbound dcbpy, O-H bend on both COOH of dcbpy
1106	1102	1106	1102	1111	sym. i.p. C-H wag on unbound dcbpy
	1021	1020		1001	Trigonal ring stretch on both bpy
	920			839	o. p. C–H wag on anchored bpy, Ti–O–Ti stretch,
	809			827	C = S stretch, dcbpy deformation, anchored COO bend
702	750			796	unsym. Ti-O-Ti stretch
	698	699		719	unsym. ring stretch on anchored bpy, O–H bend in COOH H-bond to surface
	454	512		503	Ti–O = C stretch of anchored COO grps, o.p. bpy ring deformation.
	364	397		376	Ru-NCS wag, unbound dcbpy rock
	318			320	Ru-N(bpy)_trans_ wag

## Data Availability

The data available are in Supplementary Materials.

## Supplementary Materials

The following are available online at https://www.mdpi.com/article/10.3390/nano11061491/s1. Optical spectra (Figure S1) and normal Raman spectra (Figure S2). Composition of selected excited states (Table S1), charge transfer distance, charge passed indices and oscillator strengths (Table S2), and normal mode vector displacement diagrams of vibrational frequencies (Figures S3–S6).
