# Supplementary material for: DFT and TD-DFT Investigation of a Charge Transfer Surface Resonance Raman Model of N3 Dye Bound to a Small TiO2 Nanoparticle
Source: Nanomaterials (Basel). 2021 Jun 4;11(6):1491. doi: 10.3390/nano11061491 (PMC8226483; doi:10.3390/nano11061491)
Supplement: Supplementary file 1 [file nanomaterials-11-01491-s001.zip › nanomaterials-1229597-supplementary.pdf]

Supplemental Materials

DFT and TD-DFT Investigation of a Charge Transfer Surface Resonance Raman Model of N3 Dye Bound to a Small TiO_2 Nanoparticle

Ronald L. Birke * and John R. Lombardi

Department of Chemistry and Biochemistry, The City College of the City University of New York,
160 Convent Avenue, New York, New York 10031, USA

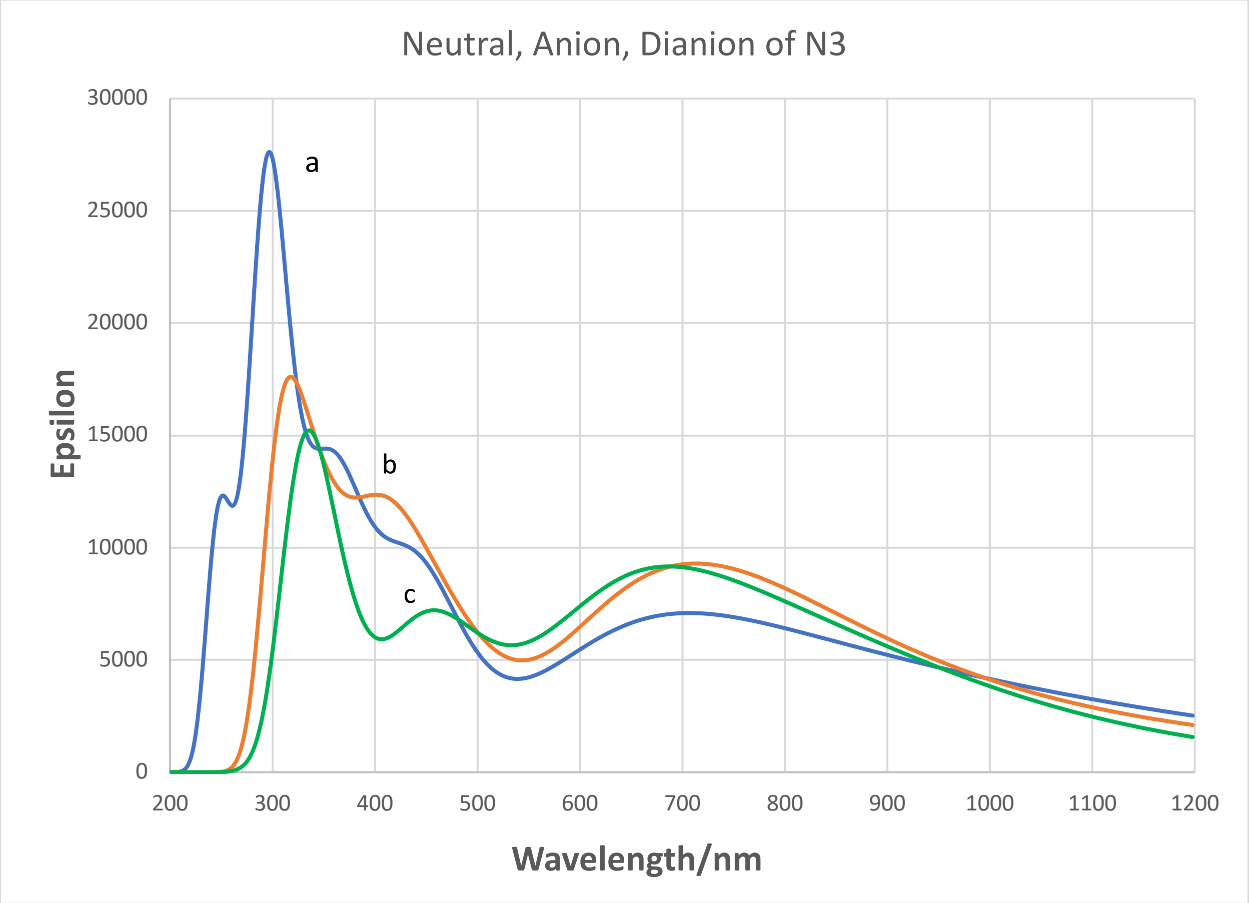

Figure S1. UV-VIS absorption spectra of various forms of N3 in vacuum with B3LYP/6-31+G(d)/LANL2DZ. FWHM broadening is 0.250 eV. a) neutral N3 blue. b) N3 anion red. c) N3 dianion (N719) green.

Table S1. Composition of Selected Excited States

Excited State 1: Singlet-A 0.9808 eV 1264.12 nm f=0.0175 <S**2>=0.000
H-3 -> L 0.11634
H-2 -> L 0.18525
H-1 -> L 0.10966
H -> L 0.64463
This state for optimization and/or second-order correction.

Excited State 2: Singlet-A 1.1057 eV 1121.36 nm f=0.0019 <S**2>=0.000
H-2 -> L -0.13440
H-1 -> L 0.67530

Excited State 3: Singlet-A 1.2227 eV 1014.00 nm f=0.0235 <S**2>=0.000
H-2 -> L -0.42492
H-1 ->L+1 0.13771
H -> L 0.18890
H -> L+1 0.49019

Excited State 4: Singlet-A 1.2770 eV 970.93 nm f=0.0151 <S**2>=0.000
H-3 -> L -0.17353
H-2 -> L 0.48513
H-1 -> L+1 0.18006
H -> L+1 0.41049
H -> L+2 0.10147

Excited State 8: Singlet-A 1.6379 eV 756.96 nm f=0.0624 <S**2>=0.000
H-3 -> L+1 0.18568
H-3 -> L+2 0.12650
H-2 -> L -0.11528
H-1 -> L+1 0.12352
H-1 -> L+2 0.19022
H -> L 0.13415
H -> L+2 0.57936

Excited State 12: Singlet-A 1.8539 eV 668.76 nm f=0.0044 <S**2>=0.000
H-3 -> L+2 0.23493
H-2 -> L+2 -0.10374
H -> L+3 0.48756
H -> L+4 0.42484

Excited State 14: Singlet-A 2.0049 eV 618.41 nm f=0.0314 <S**2>=0.000
H -> L+3 0.19488
H -> L+4 -0.15145
H -> L+5 0.62995
Excited State 16: Singlet-A 2.0711 eV 598.65 nm f=0.0020 <S**2>=0.000

H-2 -> L +3 0.50370
H-2 -> L +4 0.46029
H-2 -> L +5 -0.12585

Excited State 17: Singlet-A 2.1275 eV 582.78 nm f=0.0009 <S**2>=0.000
H-> L+3 -0.41440
H -> L+4 0.51581
H -> L+5 0.24341

Excited State 18: Singlet-A 2.1460 eV 577.76 nm f=0.0046 <S**2>=0.000
H-1 -> L+4 -0.24619
H-1 -> L+5 0.64358

Excited State 21: Singlet-A 2.2597 eV 548.68 nm f=0.0003 <S**2>=0.000
H-1 -> L+3 -0.41157
H-1 -> L+4 0.50520
H-1 -> L+5 0.25717

Table S2. Indices of Charge Transfer Distance, D_{CT} and Charge Passed, q_{CT} , and Oscillator Strengths.

State	Energy(eV)	D_{CT} (Å)	QP(a.u.)
1	0.981	3.828	0.876
2	1.106	5.394	0.914
3	1.223	4.361	0.855
4	1.277	4.274	0.87
5	1.345	4.499	0.948
6	1.431	4.573	0.893
7	1.507	5.833	0.883
8	1.638	4.897	0.905
9	1.692	5.574	0.927
10	1.72	6.069	0.934
11	1.774	4.825	0.926
12	1.854	4.409	0.921
13	1.914	5.421	0.952
14	2.005	4.923	0.91
15	2.035	4.953	0.96
16	2.071	4.373	0.956
17	2.127	10.426	0.985
18	2.146	4.804	0.957
19	2.219	4.732	0.985
20	2.253	6.526	0.952

21	2.26	10.099	0.982
22	2.35	4.647	0.932
23	2.368	5.793	0.957
24	2.389	8.21	0.914
25	2.391	4.617	0.882
26	2.445	3.485	0.843
27	2.462	11.425	0.991
28	2.513	5.107	0.955
29	2.621	5.872	0.967
30	2.621	9.478	0.977
31	2.682	4.027	0.834
32	2.709	2.688	0.788
33	2.73	5.327	0.98
34	2.747	4.587	0.88
35	2.753	8.844	0.973
36	2.815	9.584	0.996
37	2.9	11.838	0.997
38	2.945	9.081	0.998
39	2.967	11.182	0.992
40	2.989	2.935	0.793
41	3.061	4.495	0.918
42	3.093	11.979	0.999
43	3.126	11.139	0.996
44	3.134	9.517	0.996
45	3.158	11.251	0.998
46	3.201	3.932	0.845
47	3.238	1.745	0.93
48	3.265	9.042	0.997
49	3.267	8.808	0.938
50	3.273	10.393	0.991
51	3.29	3.148	0.802
52	3.316	8.854	0.992
53	3.347	3.572	0.843
54	3.357	2.143	0.676
55	3.396	3.732	0.878
56	3.4	8.564	0.975
57	3.41	11.784	0.998
58	3.422	3.122	0.862
59	3.454	8.554	0.988
60	3.476	9.859	0.995

61	3.477	11.027	0.997
62	3.484	10.575	0.992
63	3.486	2.226	0.666
64	3.497	3.235	0.83
65	3.514	3.992	0.857
66	3.531	5.036	0.862
67	3.54	6.099	0.882
68	3.558	5.91	0.869
69	3.584	4.397	0.816
70	3.589	4.083	0.846
71	3.603	9.156	0.997
72	3.611	3.235	0.656
73	3.619	10.636	0.979
74	3.648	3.559	0.661
75	3.655	8.873	0.969
76	3.662	9.654	0.986
77	3.662	4.873	0.929
78	3.668	8.58	0.973
79	3.675	4.211	0.769
80	3.684	13.133	0.996
81	3.695	3.202	0.912
82	3.714	4.408	0.982
83	3.729	8.711	0.921
84	3.744	9.003	0.858
85	3.748	5.83	0.737
86	3.756	12.262	0.999
87	3.768	6.849	0.923
88	3.775	5.75	0.937
89	3.793	9.26	0.991
90	3.795	6.609	0.905
91	3.795	5.267	0.895
92	3.799	9.525	0.969
93	3.813	6.562	0.872
94	3.82	11.491	0.998
95	3.821	7.717	0.942
96	3.832	7.35	0.941
97	3.839	12.751	0.998
98	3.854	7.191	0.868
99	3.861	9.399	0.972
100	3.866	5.017	0.798

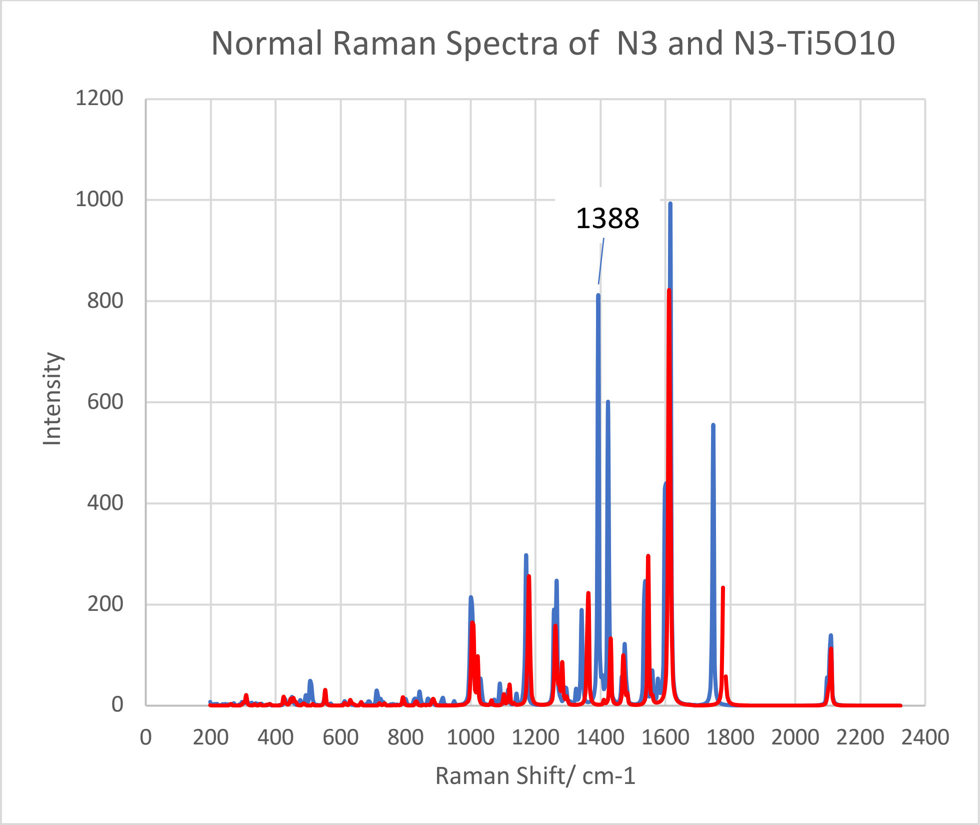

Figure S2. Raman Spectra of complex A (N3-$(TiO_2)_5$ (blue) and isolated N3 dye (red). FWHM broadening 2 cm^{-1}. A scaling factor 0.970 have been used for the Raman frequencies.

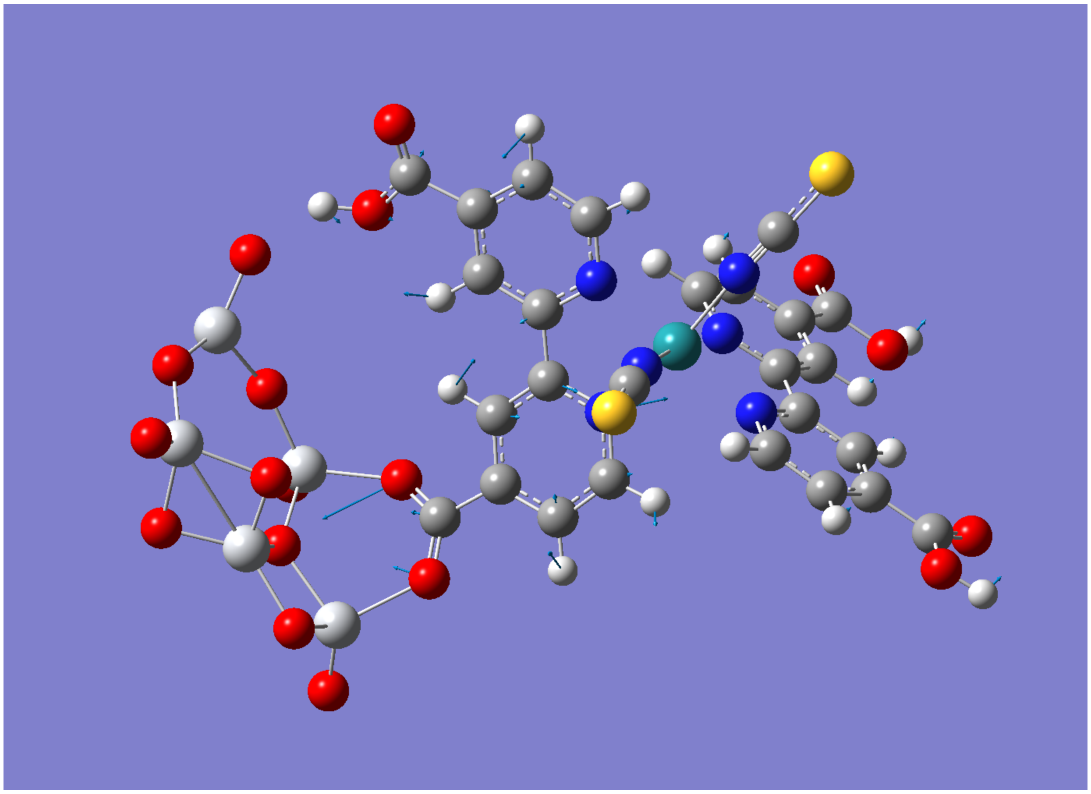

Figure S3. Vibration at scaled frequency 500.3 cm^{-1} with displacement vectors involving anchoring -COO group.

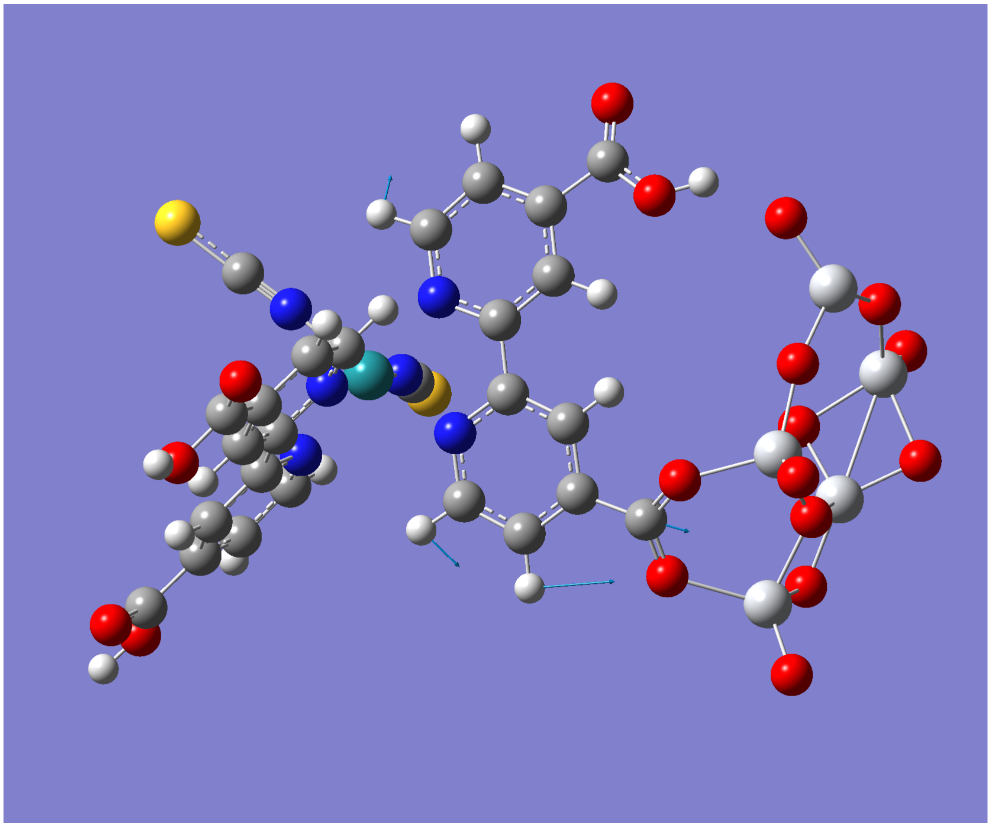

Figure S4. Vibration at scaled frequency 1388.5 cm^{-1} with displacement vectors involving anchoring -COO group.

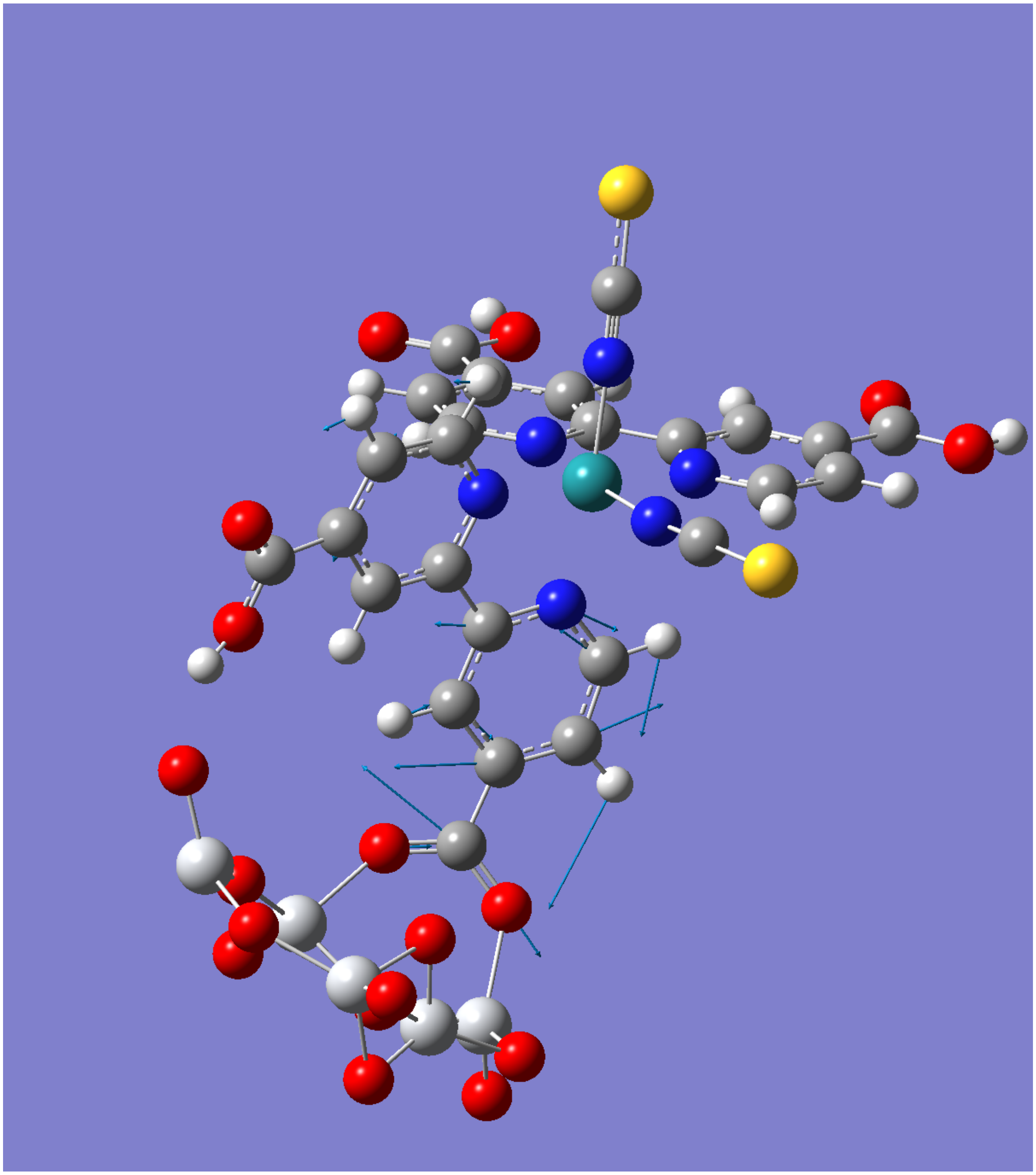

Figure S5. Vibration at scaled frequency 1534.0 cm^{-1} with displacement vectors involving anchoring -COO group.

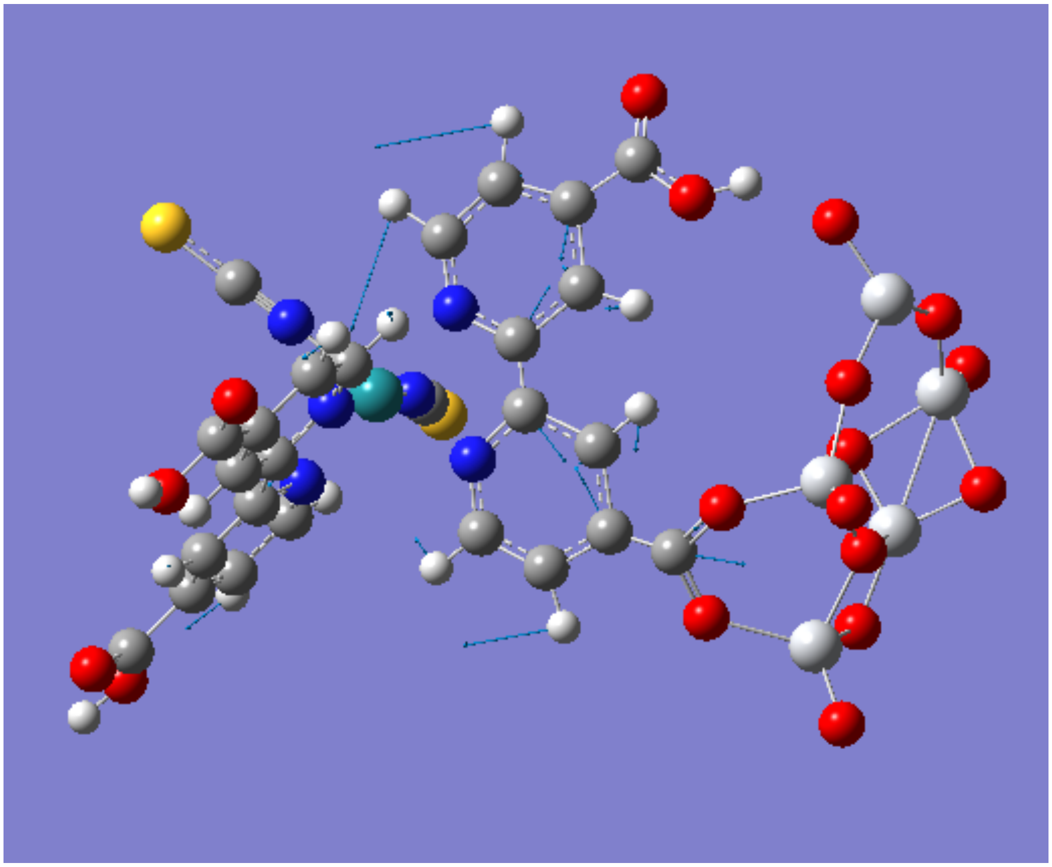

Figure S6. Vibration at scaled frequency 1420.0 cm^{-1} with displacement vectors involving anchoring -COO group.
